# Hierarchical service needs for physical fitness promotion among Urban Community older adults: a Kano model perspective

**DOI:** 10.3389/fpubh.2026.1843368

**Published:** 2026-06-22

**Authors:** Cong-Hui He, Jiao-Bao Zhao, Wen-Yun Lu, Hai-Pei Zhang

**Affiliations:** 1 Physical Education and Sports School, Soochow University, Suzhou, China; 2 School of Economics and Management, Shanghai University of Sport, Shanghai, China; 3 School of Physical Education, Shanghai University of Sport, Shanghai, China; 4 School of Physical Education, China West Normal University, Nanchong, China

**Keywords:** hierarchical needs, Kano model, older adults, physical fitness promotion, service needs

## Abstract

**Introduction:**

Physical fitness promotion services for older adults in urban communities represent a critical pathway for achieving healthy aging. While existing research has largely focused on service supply and intervention outcomes, the demand structure of older adults themselves, particularly the hierarchical differences in demand among subgroups with varying health statuses, remains insufficiently explored. This study, grounded in the Kano model, aims to identify the demand attributes of physical fitness promotion services for healthy older adults, those with chronic diseases, and those with care dependency in urban communities, to analyze the hierarchical structure and priority order of their demands, and to provide empirical evidence for optimizing community-based service provision.

**Methods:**

Based on the Kano model, this study employed the Delphi technique, questionnaire surveys, and logical analysis to examine the hierarchical structure, expectation characteristics, and dependency characteristics of physical fitness promotion service demands among older adults in urban communities. A demand map was subsequently constructed.

**Results:**

The overall hierarchical level of physical fitness promotion demands among older adults in urban communities was relatively low. The demands of healthy older adults were primarily characterized by one-dimensional and must-be quality attributes. The demands of older adults with chronic diseases were more concentrated on must-be quality attributes. The demands of older adults with care dependency further converged on basic support and care coordination services.

**Discussion:**

The allocation of service content should neither be uniformly distributed across different groups of older adults irrespective of their physical conditions and life circumstances, nor simply apply a single set of service standards. Instead, while ensuring the provision of common foundational services, targeted arrangements should be made around the most salient practical needs of each group.

## 1 Introduction

Against the backdrop of deepening population aging, the health of older adults has become a major issue with implications for both sustainable social development and the modernization of public governance (1). Existing studies suggest that health problems in later life are not manifested solely in disease itself; rather, they are also reflected in a chain of risks such as declining muscle strength, reduced balance capacity, limited mobility, and the subsequent occurrence of falls, frailty, and functional dependence (2–4). As a fundamental indicator of an individual’s physical functioning, motor capacity, and ability to perform daily activities, physical fitness is closely associated not only with older adults’ quality of life but also with their capacity for independent living and social participation (5). From this perspective, approaching older adult health governance through physical fitness promotion helps move health interventions upstream and provides a practical pathway for actively responding to population aging. To date, a substantial body of research has accumulated on physical fitness promotion among older adults, mainly in four areas. First, from the perspective of health outcomes, studies have examined the relationships among physical activity, fitness levels, sarcopenia, fall risk, activities of daily living, and the onset of disability, highlighting the fundamental role of physical fitness in maintaining functional capacity and delaying frailty in older adults (6–8). Second, from the perspective of intervention effectiveness, research has focused on the effects of resistance training, multicomponent exercise, and functional training on key fitness components in older adults, including muscle strength, balance, flexibility, and endurance (5, 9, 10). Third, from the perspective of community-based practice, scholars have paid attention to the implementation of community exercise and health-promotion services, analyzing the practical effects of comprehensive interventions on older adults’ physical functioning, health status, and overall wellbeing (11, 12). Fourth, from the perspective of healthy aging and functional capacity maintenance, researchers have explored the theoretical foundations and practical directions of integrated service provision for older adults (13–15). Overall, existing studies have paid considerable attention to the mechanisms, intervention effects, and community-based practices of physical fitness promotion among older adults, thereby demonstrating both its importance and the necessity of related service provision. However, less attention has been given to differences in the position of specific service components within older adults’ need structures. In the context of physical fitness promotion for older adults in urban communities, needs are not simply expressed in terms of whether a given service is required; rather, different service components occupy different positions within the overall structure of need, ranging from basic support to priority enhancement and value-added expansion. If analysis remains limited to general need identification, it becomes difficult to determine which services should be prioritized, which should be strengthened, and which may be gradually expanded as conditions allow. The Kano model is useful in this regard because it emphasizes that different service elements do not contribute equally to user satisfaction or perceived need. It distinguishes service components that may appear equally important on the surface into different attributes, such as must-be, one-dimensional, and attractive attributes, thereby revealing their hierarchical positions and priority order within the overall need structure. On this basis, the present study applies the Kano model to classify and analyze the hierarchical structure of needs for physical fitness promotion among different groups of older adults in urban communities, with the aim of providing a reference for determining service priorities and developing differentiated service provision.

## 2 Theoretical framework

### 2.1 The Kano model

The Kano model was proposed by Japanese scholar Noriaki Kano in the 1980s (16). Its core contribution lies in transcending traditional linear perceptions of satisfaction to reveal the nonlinear relationship between the quality attributes of a product or service and customer satisfaction. The theory classifies quality attributes into five categories. Must-be attribute (M) represents basic requirements that customers implicitly expect. When fulfilled, they do not increase satisfaction. But when absent, they lead to strong dissatisfaction. One-dimensional attribute (O) exhibits a positive linear correlation with satisfaction, the more fully such attributes are provided, the higher the level of customer satisfaction. Attractive attribute (A) manifests as features that exceed customer expectations. When provided, they enhance satisfaction, yet their absence does not cause dissatisfaction. Indifferent attribute (I) has no substantial impact on satisfaction, whether provided or not, they do not alter customer attitudes. Reverse attribute (R) reduces satisfaction as their supply increases, as they contradict customer expectations. Questionable attributes (Q) typically arise from contradictory responses or misunderstandings in questionnaire surveys, such data cannot be reasonably classified, as they neither conform to the logical relationship between satisfaction and dissatisfaction nor may reflect respondents’ misinterpretation of the questions or unclear perception of the service. Therefore, they should be excluded from empirical analysis or subjected to re-examination.

The Kano model is characterized by its use of paired positive and negative scenarios to identify need attributes. For each service item, it sets two situations—need fulfillment and need non-fulfillment—and determines the attribute type of that service item according to the combination of respondents’ reactions to these two situations, based on the Kano evaluation matrix (see Table 1). In this way, the Kano model does not identify satisfaction merely in terms of its magnitude; rather, it reveals the functional position of different service elements within the overall service system, distinguishing those that constitute indispensable basic needs, those that should be prioritized as core needs, and those that generate additional value. Following this analytical logic, the present study designed two paired questions for each physical fitness promotion service item: “How would you feel if this service were provided?” and “How would you feel if this service were not provided?” This formed a paired functional–dysfunctional question structure.

**Table 1:** Kano evaluation matrix.

Categories		Unmet needs				
		Very satisfied	Satisfied	Neutral	Dissatisfied	Very dissatisfied
Met needs	Very satisfied	Q	A	A	A	O
	Satisfied	R	I	I	I	M
	Neutral	R	I	I	I	M
	Dissatisfied	R	I	I	I	M
	Very dissatisfied	R	R	R	R	Q

It should be noted that the classic Kano questionnaire usually adopts preference- or acceptance-based response options, such as “I like it,” “I expect it,” “I am neutral,” “I can tolerate it,” and “I dislike it.” In this study, these response options were adapted into a satisfaction–dissatisfaction format, mainly in consideration of the characteristics of the respondents and the field survey context. On the one hand, the respondents were older adults aged 60 years and above, and some of them might have difficulty interpreting preference-based expressions such as “I can tolerate it” or “it should be that way.” On the other hand, physical fitness promotion services have the characteristics of both public services and health services, and older adults may more directly evaluate the presence or absence of such services in terms of satisfaction or dissatisfaction. Therefore, this study used more comprehensible satisfaction-based wording to lower the cognitive burden of the questionnaire and improve response accessibility in the field survey. However, this adaptation does not mean that the Kano questionnaire was treated as a conventional satisfaction scale. The attribute classification of each service item was still determined by the response combinations to the functional and dysfunctional questions, rather than by directly scoring the five-point response options. In other words, the five-point satisfaction-based responses mainly served to express respondents’ attitudes toward the two scenarios, while the Kano classification remained based on the paired structure of “service provided–service not provided” and the Kano evaluation matrix. At the same time, this study acknowledges that this response format differs from the preference-based options used in the classic Kano questionnaire and may influence respondents’ understanding of the dysfunctional questions, particularly among older adults. To minimize this potential influence, trained investigators provided standardized explanations of the two scenarios, namely “service provided” and “service not provided,” during the survey, and offered non-leading clarification when respondents had difficulty understanding the questions. Nevertheless, because this study did not conduct systematic cognitive interviews or comparative testing between the classic Kano options and the satisfaction-based options, the applicability of the modified response format still needs to be further validated in future research.

On the basis of attribute identification, the satisfaction–dissatisfaction coefficients were further introduced to provide a supplementary analysis of the relative influence of different need items (17). Specifically, the satisfaction coefficient (Better) reflects the extent to which satisfaction increases when a given need is fulfilled, whereas the dissatisfaction coefficient (Worse) captures the negative impact on satisfaction when that need is not fulfilled. The equations are as follows:


$$\text{Better}=\frac{A+O}{A+O+M+I}\;\text{Worse}=-\frac{O+M}{A+O+M+I}$$

In addition, to convert the qualitative classification produced by the Kano model into a quantitative priority ranking, user need sensitivity was denoted by *S*, defined as the distance from the satisfaction–dissatisfaction coordinate to the origin. Given the properties of the satisfaction–dissatisfaction framework, a larger *S* value indicates greater sensitivity and, accordingly, a stronger overall influence of that need item. The calculation is as follows:


$$S=\sqrt{{\text{Better}}^{2}+{\text{Worse}}^{2}}$$

### 2.2 Analysis of theoretical applicability

Physical fitness promotion for older adults in urban communities involves multiple service components, including physical fitness monitoring, physical fitness risk alerts, exercise intervention, and outcome feedback. These service components do not occupy the same position in older adults’ need structure. Some constitute basic services necessary for service delivery; their absence may generate dissatisfaction, whereas their provision may not necessarily produce an equivalent increase in satisfaction. Some are quality-enhancing services, for which improvements in provision are more directly associated with increased satisfaction. Others reflect additional or extended value and may create extra perceived benefits when they exceed basic expectations. A general needs assessment alone is often insufficient to reveal these hierarchical differences among service components. Compared with conventional needs assessment, satisfaction surveys, or importance ranking, the Kano model can further distinguish the attribute positions of different service items within the need structure, thereby providing a basis for identifying which services should be prioritized as basic guarantees, which should be optimized as key service components, and which may be developed as value-added services (18). Applying the Kano model to the analysis of physical fitness promotion needs among older adults in urban communities can help transform relatively abstract service needs into a concrete service structure with differentiated levels.

## 3 Research design

### 3.1 Questionnaire design

In constructing the questionnaire indicator system, this study first drew on practical cases of physical fitness promotion for older adults in urban communities, together with relevant research findings, to summarize service components and develop an initial pool of indicators. The Delphi method was then employed to organize expert consultation for the purpose of evaluating and refining the scientific validity, completeness, and clarity of the proposed indicators. A total of 10 experts participated in the consultation process, representing the fields of sports science, public health, geriatrics, and community health services, all of whom had substantial research or practical experience in relevant areas. The inclusion criteria for experts were as follows: holding an associate senior title or above, or a doctoral degree; having at least 5 years of relevant research or practical experience; being familiar with older adult health promotion, community health services, or exercise intervention; and being able to complete the consultation questionnaire independently and provide substantive comments. The expert consultation was conducted in two rounds. The first round focused on the necessity, appropriateness, and clarity of expression of the proposed indicators. Based on the feedback received, indicators were merged, removed, or revised in wording, and a second-round consultation questionnaire was subsequently developed. The second round was used to re-evaluate the revised indicators in order to improve the concentration of expert opinions and the stability of the indicator system. Expert engagement was primarily assessed by the questionnaire response rate, which was 100% in the first round and 90% in the second round. The consistency of expert opinions was evaluated comprehensively using the mean score, full-score ratio, coefficient of variation, and coordination coefficient. The criteria for retaining an indicator were set as follows: a mean importance score of at least 4.00, a coefficient of variation of no more than 0.25, and a full-score ratio of at least 0.20. Indicators that did not meet these criteria were revised or deleted in light of expert comments. After two rounds of consultation, expert opinions became more concentrated, resulting in a needs indicator system for physical fitness promotion among older adults in urban communities comprising four dimensions—physical fitness monitoring, physical fitness early warning, physical fitness intervention, and physical fitness feedback—and 16 specific indicators. The item-level Delphi results are provided in Supplementary Tables.

The physical fitness monitoring dimension focuses on the routine collection and record-keeping of physical fitness data for older adults and consists of four indicators: (1) Establishment of personal health records, referring to the creation of dynamically updated individual physical fitness health records for older adults; (2) Routine physical fitness assessment, referring to standardized physical fitness assessments conducted at least once every quarter to evaluate physical functioning; (3) Diverse testing venues, referring to the provision of flexible and convenient physical fitness monitoring services in integrated sports, medical, and eldercare settings such as community health service centers, community day-care centers, and community physical fitness health centers, thereby improving service accessibility; (4) Age-friendly testing indicators, referring to the use of assessment items suited to the functional characteristics of older adults while avoiding high-intensity or high-risk tests.

The physical fitness early warning dimension concerns the early identification and communication of physical fitness-related risks among older adults and includes four indicators: (1) Physical fitness risk alerts, referring to the generation of individualized warning messages based on multidimensional information such as physical fitness monitoring data, chronic disease history, and lifestyle factors, as well as the prediction of risks including sarcopenia, fall risk, and decline in cardiopulmonary function; (2) Interpretation of physical fitness risks, referring to the explanation of warning results by physicians or exercise health specialists, including the causes of identified risks and corresponding response strategies, in order to enhance older adults’ awareness of physical fitness-related risks; (3) Physical fitness risk grading, referring to the adoption of a four-level warning system—green (no risk), yellow (mild), orange (moderate), and red (severe)—accompanied by graded intervention recommendations; (4) Synchronization of physical fitness risks, referring to the sharing of warning information with family members or caregivers to facilitate coordinated family-based management.

The physical fitness intervention dimension focuses on improving older adults’ physical fitness through exercise-based support. It includes six indicators: (1) Personalized exercise prescription, referring to the development of individualized exercise prescriptions through an intelligent exercise prescription system based on older adults’ physical fitness assessment results and health risk levels; (2) Scientific exercise guidance, referring to the implementation of full-process exercise intervention by community exercise specialists in accordance with individualized exercise prescriptions; (3) Healthy lifestyle guidance, referring to the provision of comprehensive guidance on diet, daily routines, psychological adjustment, and related aspects; (4) Organization of social activities, referring to the regular organization of group-based social activities such as tai chi and square dancing; (5) Medical follow-up, referring to follow-up services jointly provided by community general practitioners and exercise health specialists, including health follow-up, rehabilitation training, and chronic disease management; (6) Family rehabilitation training, referring to the provision of basic exercise rehabilitation training for family members through health lectures, online courses, and similar approaches, with the aim of strengthening family caregiving capacity.

The physical fitness feedback dimension aims to achieve the continuous improvement of physical fitness promotion services through dynamic evaluation and multi-stakeholder participation. It includes two indicators: (1) Periodic reassessment, referring to quarterly reassessment of physical fitness indicators, comparison of changes before and after intervention, evaluation of intervention effects, and adjustment of subsequent plans; (2) Family involvement, referring to the involvement of family members in discussions on physical fitness feedback and the planning of subsequent exercise programs, thereby strengthening family support.

Because the Kano questionnaire is based on paired responses to functional and dysfunctional questions, its primary purpose is to identify the need attributes of service items rather than to measure a reflective latent construct. Therefore, this study assessed questionnaire quality mainly from three aspects: content validity, face validity, and response quality control. First, the indicator system was developed through a literature review, practical case analysis, and expert consultation. Experts from sports science, public health, geriatric medicine, and community health services evaluated the importance, appropriateness, completeness, and operability of the indicators, and provided suggestions for item deletion, merging, renaming, and wording revision, thereby strengthening the content validity of the questionnaire. Second, in terms of item wording, the research team revised professional, abstract, or conceptually ambiguous expressions based on expert feedback, and sought to translate service content into concrete service scenarios that older adults could more easily understand. This process was intended to improve the face validity of the questionnaire and the comprehensibility of the items for respondents. Third, during the field survey, trained investigators provided standardized explanations of the two scenarios, namely “service provided” and “service not provided,” as well as the response procedure. For respondents with reading or comprehension difficulties, investigators provided non-leading explanations, and, when necessary, accompanying caregivers assisted in confirming relevant information. Finally, after the questionnaires were collected, the research team checked response completeness and logical consistency. The questionable attribute (Q) in the Kano model was also used to identify potential comprehension difficulties or response contradictions. Questionnaires with substantial missing responses on key items, abnormal response patterns, or clear logical inconsistencies were excluded.

### 3.2 Questionnaire survey

This study adopted a questionnaire survey design combining multi-city, multi-setting stratified site selection with on-site convenience sampling. The purpose of the study was not to obtain a population-representative sample of urban older adults through strict probability sampling, but to examine the attribute structure and group differences of older adults’ physical fitness promotion service needs across different cities and community service-related settings. Accordingly, the “urban community older adults” referred to in this study should be more accurately defined as older adults recruited from urban community service-related sites. The findings are therefore intended to reveal the structural characteristics of physical fitness promotion service needs among older adults in such service-contact contexts, and should not be directly generalized to all community-dwelling older adults in urban areas. Regarding city selection, Shanghai, Zhengzhou in Henan Province, and Chengdu in Sichuan Province were selected as the survey cities, taking into account regional differences in urban development, community health service resources, and the foundation of service provision for older adults across eastern, central, and western China. Shanghai represented the eastern region, Zhengzhou the central region, and Chengdu the western region. Regarding survey sites, residential communities, community health service centers, community day-care centers, community physical fitness and health centers, eldercare institutions, universities for older adults, and hospitals were selected based on the main settings in which older adults encounter daily living support, health management, exercise participation, and care services. Although these sites differ in functional attributes, target populations, and service content, they are all important settings in which older adults may access physical fitness promotion, health management, exercise guidance, or care-related support services. They therefore helped cover older adults with different health conditions, functional levels, and degrees of service dependence.

During the field survey, eligible older adults aged 60 years and above were invited to complete the questionnaire. Participants were required to be long-term residents of the survey cities, have basic communication and comprehension ability, and be able to complete the questionnaire independently or with assistance from trained investigators. Respondents recruited from different survey sites were not treated as separate populations, nor were they regarded as a representative sample of all urban community-dwelling older adults. Rather, they were considered older respondents in urban community service-related settings, and were used to analyze the attribute structure and stratified characteristics of their physical fitness promotion service needs. Given the high heterogeneity of older adults in urban community contexts in terms of health status, functional level, and care needs, analyzing older adults as a single homogeneous group would make it difficult to accurately identify differences in physical fitness promotion needs and to inform stratified service provision. Drawing on the World Health Organization’s World Report on Ageing and Health published in 2015 (14), this study classified urban community older adults into three groups according to health status and functional status: older adults without chronic diseases or care dependency, older adults with chronic diseases, and older adults with care dependency. This classification provided a stratified analytical framework for examining physical fitness promotion needs. In the specific grouping process, chronic disease status was determined primarily based on respondents’ self-reported physician diagnoses. The questionnaire asked whether respondents had any physician-diagnosed chronic disease. Those who clearly reported having one or more physician-diagnosed chronic diseases were included in the chronic disease-related classification. Because this study focused on the influence of chronic disease status on the attribute structure of physical fitness promotion service needs, rather than on differences in service needs across specific chronic disease types, the questionnaire did not systematically collect detailed information on major chronic disease types, disease duration, or multimorbidity. Care dependency was assessed with reference to validated ADL items commonly used in older adult capacity assessments in China, particularly the national standard Specification for Ability Assessment of Older Adults (GB/T 42195-2022). The assessment focused on basic daily activities such as eating, dressing, toileting, indoor mobility, and bathing. Older adults themselves or their accompanying caregivers reported their usual ability to perform these activities. If an older adult needed assistance with one or more basic daily activities, this indicated limitation in ADL and the respondent was classified into the care dependency group. In the final classification, this study followed a function-first principle. Respondents who met the criterion for ADL limitation were classified into the care dependency group regardless of whether they also had chronic diseases. Among those not classified as care-dependent, respondents who reported one or more physician-diagnosed chronic diseases were classified into the chronic disease group. Respondents who did not report a diagnosed chronic disease and did not meet the criterion for ADL limitation were classified into the group without chronic diseases or care dependency.

The survey was conducted from May to September 2025. A total of 1,953 questionnaires were distributed, of which 1,688 valid responses were returned, yielding an effective response rate of 86.43%. Among the 1,688 valid questionnaires, 608 respondents were classified as healthy older adults, 992 as older adults with chronic diseases, and 88 as older adults with care dependency. The demographic characteristics of the three groups are presented in Table 2.

**Table 2:** Demographic characteristics of the three groups of older adults (*N* = 1,688).

Variable	Category	Healthy older adults (*N* = 608)	Older adults with chronic diseases (*N* = 992)	Older adults with care dependency (*N* = 88)
Gender	Female = 0	52.92%	55.59%	48.86%
	Male = 1	47.08%	44.41%	51.14%
Age	60–70 years old = 0	49.09%	52.80%	13.64%
	71–80 years = 1	36.59%	33.88%	38.64%
	81–90 years = 2	13.81%	13.32%	47.73%
	90 years and over = 3	0.51%	0.00%	0.00%
Living arrangements	Living alone = 0	4.33%	17.76%	0.00%
	Living with spouse/partner = 1	45.97%	42.60%	11.36%
	Living with children (or other relatives) = 2	34.88%	29.28%	9.09%
	Living in a care home or nursing home = 3	14.82%	10.36%	79.55%
Marital status	No spouse = 0	29.54%	32.07%	26.14%
	Married/in a relationship = 1	70.46%	67.93%	73.86%
Level of education	Lower secondary education or below = 0	44.25%	42.93%	35.23%
	Secondary school = 1	53.12%	52.63%	55.68%
	Undergraduate degree or above = 2	2.62%	4.44%	9.09%
Employment status	Unemployed = 0	84.17%	89.34%	100.00%
	In employment = 1	15.83%	10.66%	0.00%
Pension insurance	No pension insurance = 0	21.98%	19.41%	22.73%
	With pension insurance = 1	78.02%	80.59%	77.27%
Region	East = 0	34.78%	29.11%	31.82%
	Central = 1	31.45%	25.49%	29.55%
	West = 2	33.77%	45.40%	38.63%

## 4 Hierarchical structure of physical fitness promotion needs among older adults in urban communities

### 4.1 Physical fitness promotion needs of healthy older adults

#### 4.1.1 Overall hierarchical structure of needs among healthy older adults

Analysis of the collected data revealed that 3 types of needs were classified as attractive attributes (A), 4 as one-dimensional attributes (O), 4 as must-be attributes (M), 4 as indifferent attributes (I), and 1 as a reverse attribute (R), as detailed in Table 3.

**Table 3:** Kano attribute classification of physical fitness promotion needs among healthy older adults.

Service	A	O	M	I	R	Q	Kano classification
Establishment of personal health records	15.30%	12.83%	49.67%	19.24%	1.97%	0.99%	M
Routine physical fitness assessment	14.14%	8.22%	60.53%	13.16%	2.96%	0.99%	M
Diverse testing venues	48.03%	11.18%	8.22%	18.09%	4.93%	9.55%	A
Age-friendly testing indicators	33.88%	6.41%	13.49%	44.08%	1.32%	0.82%	I
Physical fitness risk alerts	16.78%	40.30%	18.09%	13.32%	7.73%	3.78%	O
Interpretation of physical fitness risks	10.86%	46.88%	20.56%	19.24%	1.97%	0.49%	O
Physical fitness risk grading	20.23%	38.49%	19.24%	14.63%	4.61%	2.80%	O
Synchronization of physical fitness risks	20.72%	11.18%	22.04%	41.78%	1.97%	2.30%	I
Personalized exercise prescription	15.63%	16.12%	42.93%	14.97%	6.58%	3.78%	M
Scientific exercise guidance	55.59%	12.83%	10.53%	19.08%	0.99%	0.98%	A
Healthy lifestyle guidance	39.47%	11.68%	18.09%	27.80%	0.99%	1.97%	A
Organization of social activities	16.61%	37.34%	21.71%	15.79%	2.96%	5.59%	O
Medical follow-up	31.91%	6.41%	12.83%	43.42%	3.62%	1.81%	I
Family rehabilitation training	18.42%	1.97%	32.57%	43.58%	2.14%	1.32%	I
Periodic reassessment	15.30%	10.20%	50.65%	18.91%	2.80%	2.14%	M
Family involvement	7.07%	6.25%	10.53%	34.70%	39.31%	2.14%	R

A: attractive attribute, O: one-dimensional attribute, M: must-be attribute, I: indifferent attribute, R: reverse attribute, Q: questionable attribute; the same applies below.

The results indicate that healthy older adults showed a clear hierarchical structure in their physical fitness promotion needs. The establishment of personal health records, routine physical fitness assessment, personalized exercise prescription, and periodic reassessment were generally identified as must-be attributes. This suggests that healthy older adults have developed basic expectations for continuous and systematic health management services. Meeting these needs is a prerequisite for maintaining service satisfaction, whereas their absence may substantially reduce overall evaluations. Physical fitness risk alerts, interpretation of physical fitness risks, physical fitness risk grading, and organization of social activities were classified as one-dimensional attributes. The first three items collectively point to a strong need for informational support, suggesting that healthy older adults are concerned not only with “what has been measured,” but also with “what the results mean.” Physical fitness promotion therefore needs to combine assessment with health education. The expectation for organization of social activities further suggests that older adults’ service evaluations extend beyond physiological improvement alone and also involve social participation and emotional support.

Diverse testing venues, scientific exercise guidance, and healthy lifestyle guidance were classified as attractive attributes. Although these needs are not essential for maintaining service satisfaction, their provision can substantially enhance older adults’ service experience and willingness to participate, and may also contribute to the routinization of health-related behaviors. Accordingly, once must-be and one-dimensional attributes have been adequately secured, the moderate introduction of attractive attributes may help create differentiated service advantages and strengthen healthy older adults’ intrinsic motivation to engage in physical fitness promotion over the longer term.

In contrast, age-friendly testing indicators, synchronization of physical fitness risks, medical follow-up, and family rehabilitation training were generally classified as indifferent attributes. This suggests that, in the context of relatively preserved self-care ability, healthy older adults may still focus primarily on self-management, while showing relatively limited sensitivity to extended support services. Family involvement was identified as a reverse attribute. This may indicate that, among healthy older adults with stronger self-care ability, some respondents tended to maintain autonomy in health management and had relatively low need for family involvement. It may also be related to some older adults’ reluctance to increase the care burden on family members. Overall, the physical fitness promotion needs of healthy older adults can be summarized as a hierarchical pattern in which basic guarantee services constitute the core, functional extension services provide added value, and value-added experience services serve as motivational enhancers. In practice, the effective provision of must-be and one-dimensional attributes may be prioritized, while attractive attributes may be selectively introduced when resources allow, so as to enhance the sustainability of physical fitness promotion behaviors and improve participation motivation.

#### 4.1.2 Satisfaction-dissatisfaction coefficient analysis of physical fitness promotion needs among healthy older adults

To more comprehensively analyze the physical fitness promotion needs of older adults in urban communities, this study further employs the satisfaction-dissatisfaction coefficient analysis method proposed by Berger et al. (19). This method quantifies the positive and negative effects of service function fulfillment on the satisfaction of older adults (as shown in Table 4). A higher satisfaction coefficient indicates that the service function has a stronger effect on enhancing the satisfaction of older adults, whereas a larger absolute value of the dissatisfaction coefficient suggests that the absence of the function exerts a greater negative impact on their satisfaction. The satisfaction-dissatisfaction coefficient analysis allows for further differentiation among the physical fitness promotion need items of older adults. Additionally, the priority of each need indicator is determined to clarify the order of importance. Let “*S*” represent the sensitivity of each need, with a larger “*S*” value indicating higher sensitivity and greater impact.

**Table 4:** Satisfaction–dissatisfaction coefficients of physical fitness promotion needs among healthy older adults.

Service	Attribute	Satisfaction coefficient	Dissatisfaction coefficient	Sensitivity
Establishment of personal health records	M	0.290	-0.644	0.706
Routine physical fitness assessment	M	0.233	-0.716	0.753
Diverse testing venues	A	0.692	-0.227	0.729
Age-friendly testing indicators	I	0.412	−0.203	0.459
Physical fitness risk alerts	O	0.645	−0.660	0.923
Interpretation of physical fitness risks	O	0.592	−0.691	0.910
Physical fitness risk grading	O	0.634	−0.623	0.889
Synchronization of physical fitness risks	I	0.333	−0.347	0.481
Personalized exercise prescription	M	0.354	−0.659	0.748
Scientific exercise guidance	A	0.698	−0.238	0.738
Healthy lifestyle guidance	A	0.527	−0.307	0.610
Organization of social activities	O	0.590	−0.646	0.875
Medical follow-up	I	0.405	−0.203	0.453
Family rehabilitation training	I	0.211	−0.358	0.415
Periodic reassessment	M	0.268	−0.640	0.694
Family involvement	R	0.228	−0.287	0.366

**Figure 1:**
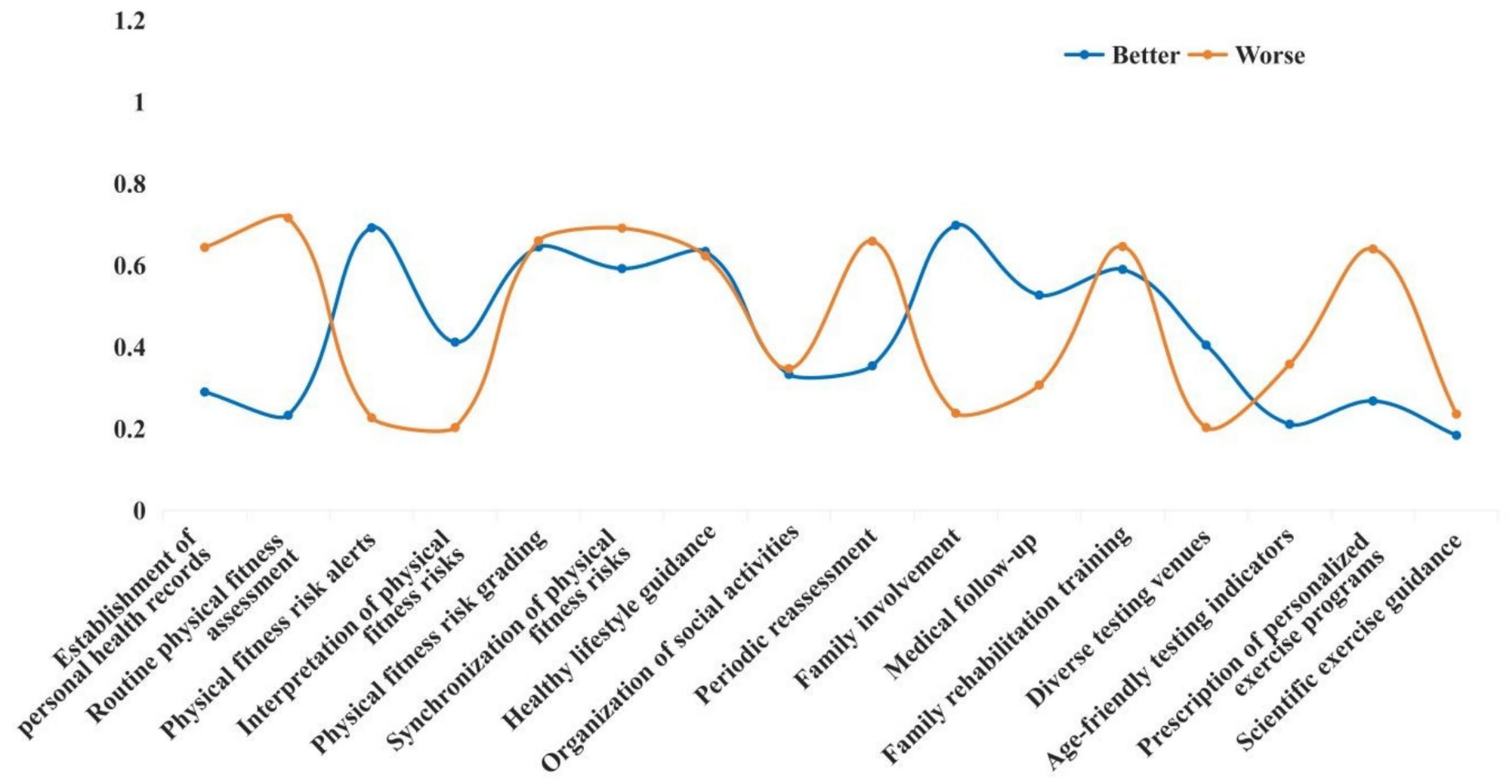
Satisfaction–dissatisfaction coefficients of each service item for healthy older adults.

As shown in Figure 1, satisfaction and dissatisfaction coefficients varied considerably across service items among healthy older adults, indicating that the positive and negative effects of different service items on the satisfaction of healthy older adults in the community differ depending on whether the service is fulfilled or not.

More specifically, the three service items with the highest satisfaction coefficients were scientific exercise guidance (0.698), diverse testing venues (0.692), and physical fitness risk alerts (0.645). This suggests that, once basic service provision has been secured, further optimization and innovation in these areas can generate a pronounced gain effect, making them critical levers for creating experiences that exceed expectations and for enhancing healthy older adults’ sense of benefit and wellbeing. Among them, scientific exercise guidance, by offering individualized solutions, directly addresses healthy older adults’ central concern with how to exercise effectively while ensuring safety. It is also highly consistent with the policy orientation of the Opinions of the State Council on Implementing the Healthy China Initiative, which emphasizes strengthening scientific fitness guidance services, and thus provides an important basis for community-level resource allocation and service optimization.

By contrast, the dissatisfaction coefficient reflects the loss effect associated with service absence. The findings show that service items with relatively large absolute dissatisfaction coefficients were concentrated in physical fitness risk alerts (−0.660), interpretation of physical fitness risks (−0.691), physical fitness risk grading (−0.623), organization of social activities (−0.646), and personalized exercise prescription (−0.659). A possible explanation is that, with rising health awareness and improved access to health information, the timely acquisition of health risk information, understanding its implications, and receiving targeted intervention recommendations have gradually been internalized as basic expectations of health management among healthy older adults. Once such services are absent, their trust in community-based physical fitness promotion and their willingness to participate are likely to be weakened.

A further synthesis of the satisfaction coefficients, dissatisfaction coefficients, and the resulting sensitivity values (*S*) shows that physical fitness risk alerts (*S* = 0.923), interpretation of physical fitness risks (*S* = 0.910), and physical fitness risk grading (*S* = 0.889) ranked among the highest in sensitivity. Because these services can both substantially enhance satisfaction and markedly intensify dissatisfaction when absent, they may be regarded as priority items for maintaining the effectiveness of the overall physical fitness promotion system. In contrast, medical follow-up, family rehabilitation training, age-friendly testing indicators, family involvement, and synchronization of physical fitness risks displayed relatively low sensitivity values (*S* < 0.5). Their direct contribution to healthy older adults’ satisfaction with health services appears limited, which may be related to this group’s relatively strong self-care capacity and independent living ability, making them less sensitive to the marginal value of such services.

**Figure 2:**
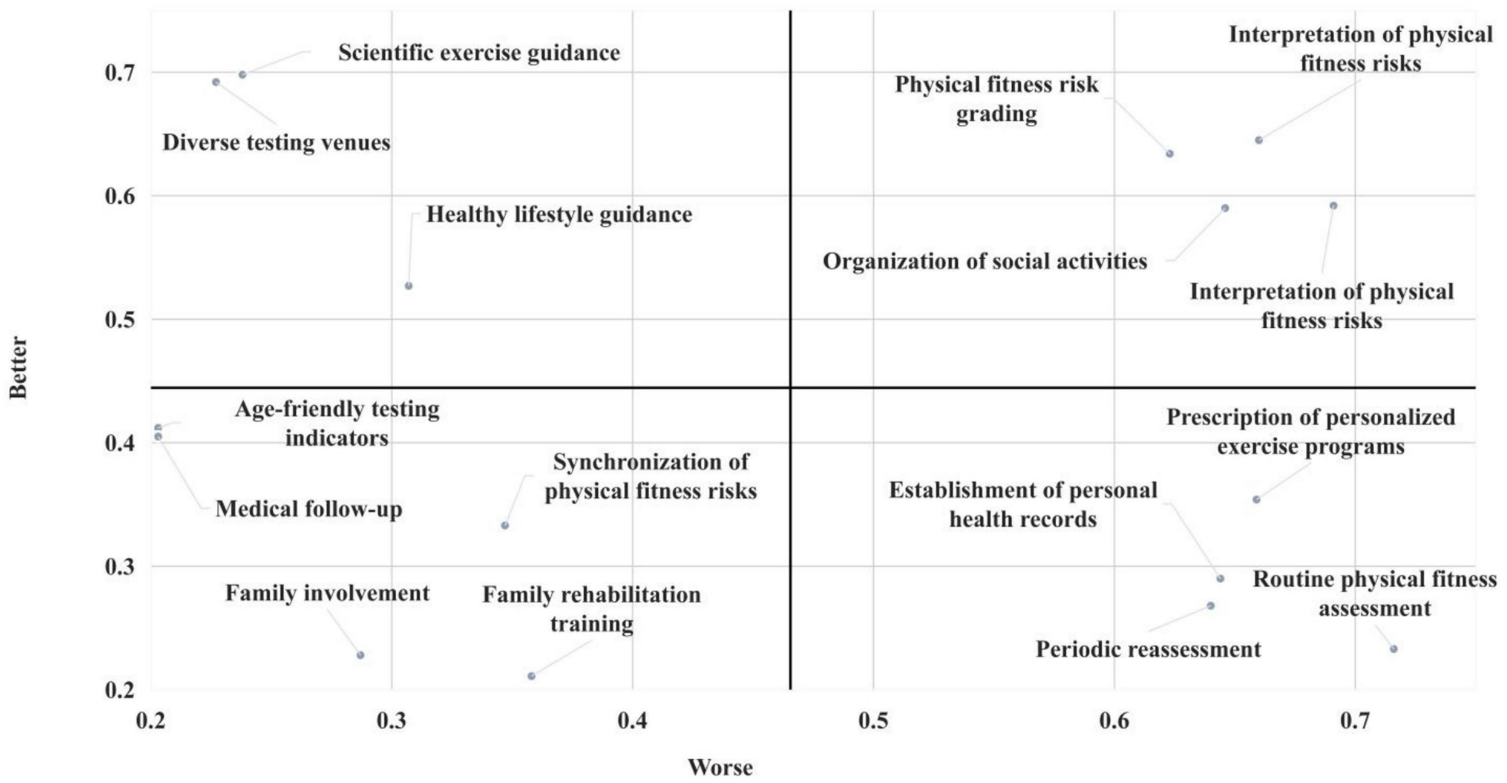
Satisfaction–dissatisfaction coefficient matrix of physical fitness promotion service needs among healthy older adults.

At the same time, plotting the absolute values of the satisfaction and dissatisfaction coefficients in a scatter diagram (see Figure 2) provides an intuitive presentation of the position of each service item within the need-sensitivity matrix. The first quadrant represents one-dimensional attributes, the second quadrant represents attractive attributes, the third quadrant corresponds to indifferent and reverse attributes, and the fourth quadrant corresponds to must-be attributes. The horizontal axis represents the dissatisfaction coefficient: the farther to the right a service item is located, the greater the dissatisfaction caused by its absence. The vertical axis represents the satisfaction coefficient: the higher a service item is located, the stronger its contribution to satisfaction when provided.

Finally, based on the preceding data analysis, the priority order of service items for healthy older adults was determined. From the perspective of public service guarantee, the ranking criteria incorporated both quantitative and qualitative dimensions. First, as a composite indicator derived from the satisfaction–dissatisfaction analysis, the S value quantitatively captures the combined sensitivity of each service item to both the satisfaction-enhancing effect associated with improved provision (satisfaction coefficient) and the dissatisfaction-inducing effect associated with service absence (dissatisfaction coefficient). It can therefore be used to identify, from the perspective of potential benefit, those services with the greatest intervention value. Second, the long-standing application of the Kano model has shown that different need attributes occupy different strategic positions within a service system: the absence of must-be needs directly leads to pronounced dissatisfaction and thus requires priority in service provision; one-dimensional needs have a positive linear relationship with satisfaction and constitute the core of service optimization; the fulfillment of attractive needs creates an additional sense of delight, although their absence has only limited negative consequences; and indifferent needs exert relatively weak influence on satisfaction under the current circumstances (20). Accordingly, in determining the priority order of service provision, this study first followed the attribute hierarchy of must-be > one-dimensional > attractive > indifferent, and then, within each attribute category, established the internal ranking of service items by combining their Kano classification results with the magnitude of their S values (see Table 5).

**Table 5:** Priority ranking of need indicators by Kano attribute category among healthy older adults.

Need attribute	Priority intervention ranking of need indicators
Must-be attribute	Routine physical fitness assessment > Personalized exercise prescription > Establishment of personal health records > Periodic reassessment
One-dimensional attribute	Physical fitness risk alerts > Interpretation of physical fitness risks > Physical fitness risk grading > Organization of social activities
Attractive attribute	Scientific exercise guidance > Diverse testing venues > Healthy lifestyle guidance
Indifferent attribute	Synchronization of physical fitness risks > Age-friendly testing indicators > Medical follow-up > Family rehabilitation training
Reverse attribute	Family involvement

Overall, the needs of healthy older adults exhibit a hierarchical structure characterized by basic support, functional expansion, and value-added experience. Within this structure, must-be attributes such as personal health records, physical fitness assessment, and exercise prescriptions constitute the foundation of the service system. One-dimensional attributes, including physical fitness risk alerts, interpretation of physical fitness risks, and social activities, serve as the core drivers of satisfaction enhancement. Attractive attributes, such as scientific exercise guidance and diversified assessment options, represent the main entry points for creating experiences that exceed expectations and for stimulating active participation.

### 4.2 Physical fitness promotion needs of older adults with chronic diseases

#### 4.2.1 Overall hierarchical structure of needs among older adults with chronic diseases

Using SPSS 21.0 statistical analysis software, the collected data were analyzed. The results revealed that 4 types of needs were classified as attractive attributes (A), 2 as one-dimensional attributes (O), 8 as must-be attributes (M), 2 as indifferent attributes (I), and 0 as a reverse attribute (R), as detailed in Table 6.

**Table 6:** Kano attribute classification of physical fitness promotion needs among older adults with chronic diseases.

Function/service	A	O	M	I	R	Q	Kano classification
Establishment of personal health records	11.29%	11.09%	46.47%	21.57%	5.04%	4.54%	M
Routine physical fitness assessment	12.70%	8.37%	55.44%	13.41%	5.04%	5.04%	M
Diverse testing venues	51.21%	8.57%	9.27%	16.13%	6.15%	8.67%	A
Age-friendly testing indicators	45.06%	11.19%	10.89%	17.54%	6.65%	8.67%	A
Physical fitness risk alerts	17.64%	13.91%	47.78%	16.23%	3.33%	1.11%	M
Interpretation of physical fitness risks	14.42%	12.20%	36.99%	27.52%	3.63%	5.24%	M
Physical fitness risk grading	14.62%	14.42%	43.14%	18.35%	6.14%	3.33%	M
Synchronization of physical fitness risks	17.44%	10.38%	20.06%	37.00%	14.01%	1.11%	I
Personalized exercise prescription	9.68%	10.48%	57.76%	15.83%	4.44%	1.81%	M
Scientific exercise guidance	14.62%	16.23%	49.18%	14.52%	3.33%	2.12%	M
Healthy lifestyle guidance	14.52%	35.58%	17.34%	15.22%	9.78%	7.56%	O
Organization of social activities	9.68%	37.40%	18.75%	24.49%	7.46%	2.22%	O
Medical follow-up	62.60%	4.33%	10.08%	21.57%	1.42%	0.00%	A
Family rehabilitation training	10.79%	23.39%	15.32%	34.68%	11.28%	4.54%	I
Periodic reassessment	9.17%	8.97%	55.04%	20.26%	3.33%	3.23%	M
Family involvement	29.13%	12.30%	12.00%	23.18%	21.07%	2.32%	A

The service needs of older adults with chronic diseases for physical fitness promotion exhibit structural characteristics that differ from those of healthy older adults, most notably in their stronger orientation toward risk management, greater service rigidity, and more pronounced need for continuity. The findings show that the establishment of personal health records, routine physical fitness assessment, physical fitness risk alerts, interpretation of physical fitness risks, physical fitness risk grading, personalized exercise prescription, scientific exercise guidance, and periodic reassessment were all classified as must-be attributes, forming the rigid foundation of their need structure. The absence of any one of these components may disrupt the chain of health management, not only lowering service satisfaction but also weakening the effectiveness of chronic disease control and functional maintenance.

Diverse testing venues, age-friendly testing indicators, medical follow-up, and family involvement were classified as attractive attributes, reflecting that, beyond basic service requirements, older adults with chronic diseases also express higher-level needs for service convenience, human-centered design, sustained social support, and emotional companionship (21). Healthy lifestyle guidance and organization of social activities were identified as one-dimensional attributes, representing key components in shifting chronic disease management from a focus on disease control toward a broader orientation of health promotion. By contrast, synchronization of physical fitness risks and family rehabilitation training were classified as indifferent attributes, indicating a relatively limited effect on satisfaction.

Overall, the service needs of older adults with chronic diseases for physical fitness promotion may be summarized as a three-tier structure of rigid foundation, experience enhancement, and functional supplementation. This structure places emphasis not only on disease risk management and basic treatment, but also on improvements in quality of life and social integration. Under conditions of resource constraint, priority may be given to ensuring the stable provision of must-be attributes, while attractive and one-dimensional attributes may serve as important entry points for enhancing service engagement and improving intervention outcomes, thereby supporting the gradual refinement of the service system.

#### 4.2.2 Satisfaction-dissatisfaction coefficient analysis of physical fitness promotion needs among older adults with chronic diseases

Table 7 summarizes the satisfaction coefficients, dissatisfaction coefficients, and sensitivity indicators for each service element related to physical fitness promotion among older adults with chronic diseases. Based on these results, the degree of influence of different service elements on changes in satisfaction among older adults with chronic diseases is depicted, thereby identifying the key drivers and allocation priorities for physical fitness promotion services for this population.

**Table 7:** Satisfaction–dissatisfaction coefficients of physical fitness promotion needs among older adults with chronic diseases.

Service	Attribute	Satisfaction coefficient	Dissatisfaction coefficient	Sensitivity
Establishment of personal health records	M	0.247	−0.637	0.683
Routine physical fitness assessment	M	0.234	−0.710	0.747
Diverse testing venues	A	0.702	−0.209	0.732
Age-friendly testing indicators	A	0.664	−0.261	0.714
Physical fitness risk alerts	M	0.330	−0.646	0.725
Interpretation of physical fitness risks	M	0.292	−0.540	0.614
Physical fitness risk grading	M	0.321	−0.636	0.712
Synchronization of physical fitness risks	I	0.328	−0.359	0.486
Personalized exercise prescription	M	0.215	−0.728	0.759
Scientific exercise guidance	M	0.326	−0.692	0.765
Healthy lifestyle guidance	O	0.606	−0.640	0.882
Organization of social activities	O	0.521	−0.622	0.811
Medical follow-up	A	0.679	−0.146	0.695
Family rehabilitation training	I	0.406	−0.460	0.613
Periodic reassessment	M	0.194	−0.685	0.712
Family involvement	A	0.541	−0.317	0.627

**Figure 3:**
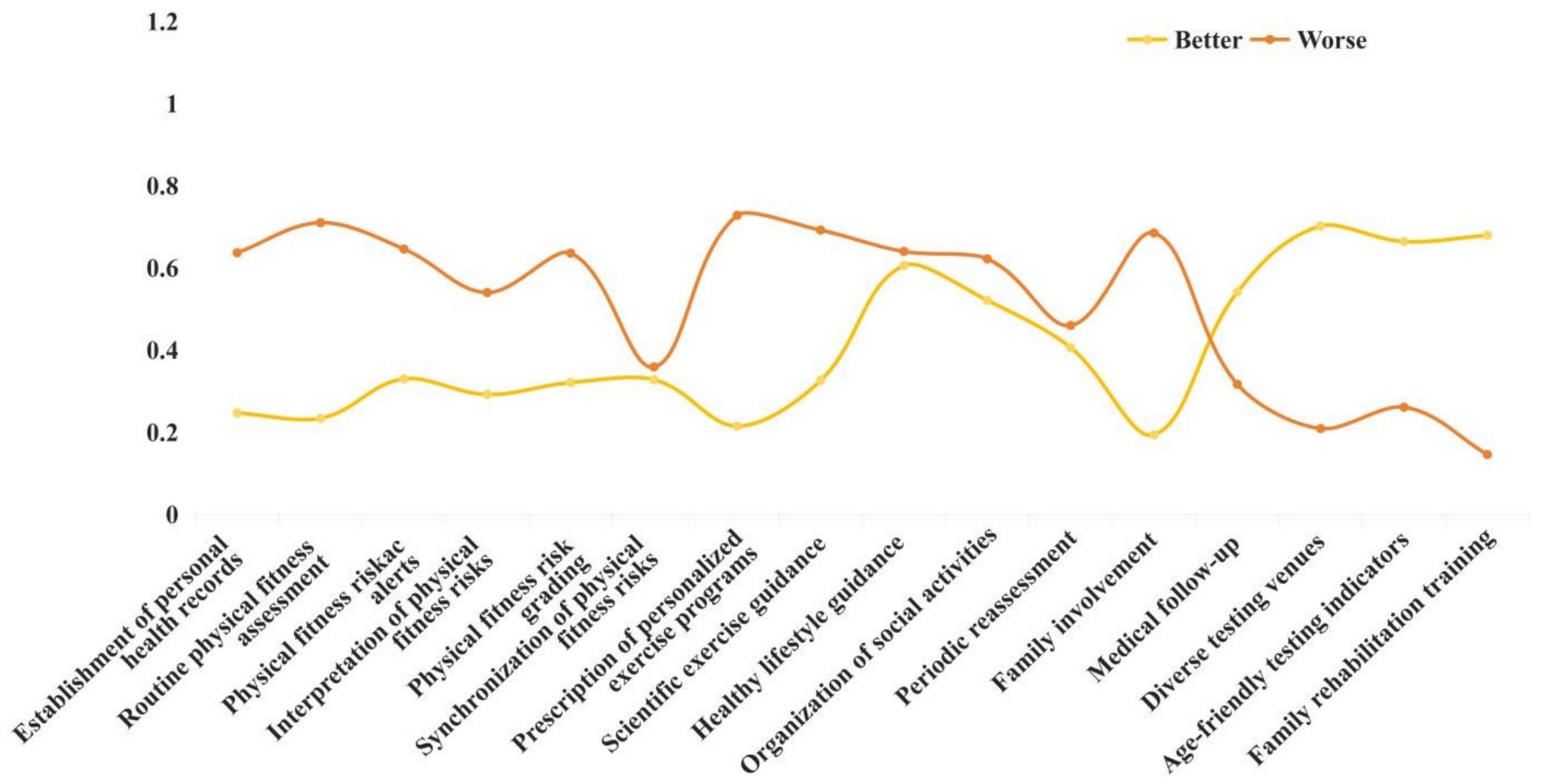
Satisfaction–dissatisfaction coefficients of each service item for older adults with chronic diseases.

Overall, the physical fitness promotion service needs of older adults with chronic diseases showed a stronger dependency orientation than expectation orientation, as shown in Figure 3. These services appear to be perceived less as optional improvement-oriented inputs and more as baseline components of community health services for older adults with chronic diseases. This may be related to their long-term needs for disease management, functional maintenance, and health risk prevention. Compared with healthy older adults, older adults with chronic diseases may place greater emphasis on continuous assessment, physical fitness risk alerts, professional guidance, and lifestyle management. As a result, their service evaluations may be more strongly affected by whether these basic services are adequately provided. First, as disease duration increases and self-management experience accumulates, individuals may shift from passive coping to more active attention to their own health status, thereby increasing their dependence on professional services. Second, with the continued advancement of the Healthy China strategy, older adults with chronic diseases may have higher normative expectations for physical fitness promotion services, viewing them as basic support for maintaining physical function and delaying disease progression rather than as additional welfare (22). Third, the desire to maintain self-care ability and family role independence may further reinforce their reliance on sustainable professional services. The relatively high dissatisfaction coefficients suggest that older adults with chronic diseases may be more sensitive to the absence or insufficient provision of relevant services. Therefore, in service allocation, priority may be given to the continuous provision of basic services such as routine assessment, physical fitness risk alerts, periodic reassessment, and personalized exercise prescription, while the quality of experience-enhancing services may be gradually improved on this basis.

Specifically, the satisfaction coefficients showed that diverse testing venues (0.702), age-friendly testing indicators (0.664), and medical follow-up (0.679) had relatively strong positive effects on service experience. Diverse testing venues can reduce temporal and spatial costs and improve service accessibility. Age-friendly testing indicators reflect the professionalism and specificity of services, which may help older adults feel understood and respected. Medical follow-up can provide continuous professional support and safety assurance, and may enhance older adults’ perceptions of service continuity and professionalism. In contrast, the dissatisfaction coefficients indicated that the absence or insufficient provision of services such as routine physical fitness assessment (−0.710), physical fitness risk alerts (−0.646), and periodic reassessment (−0.685) would have particularly pronounced negative effects. This reflects their irreplaceable role as rigid foundational services in chronic disease self-management and risk prevention. If these services are not provided consistently, service satisfaction may be directly reduced, and the effectiveness of health management may also be weakened.

**Figure 4:**
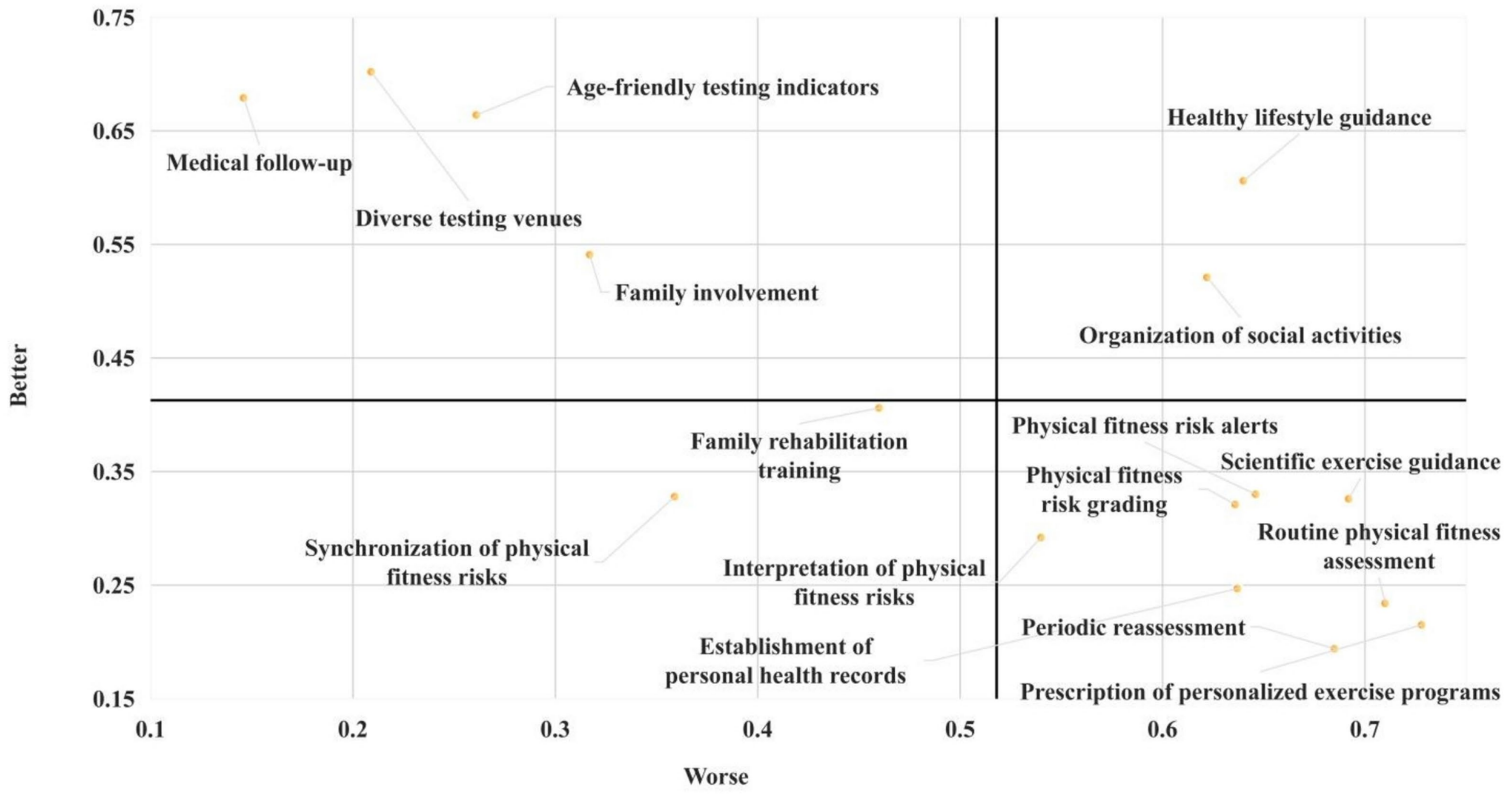
Satisfaction–dissatisfaction coefficient matrix of physical fitness promotion service needs among older adults with chronic diseases.

The composite sensitivity values further reveal the combined influence of different services on both satisfaction and dissatisfaction. The findings show that services with relatively high sensitivity—such as scientific exercise guidance, healthy lifestyle guidance, and organization of social activities—function as key performance drivers shaping the overall evaluations of older adults with chronic diseases and may therefore be prioritized for quality improvement once basic services are secured. By contrast, items such as synchronization of physical fitness risks and family rehabilitation training, which display relatively low sensitivity, are more appropriately positioned as supplementary services. Using satisfaction coefficients and dissatisfaction coefficients as the vertical and horizontal axes, respectively, and dividing the space into four quadrants based on their mean values (see Figure 4), the functional positioning of each service element can be presented more clearly in terms of whether it may be prioritized for basic guarantee, targeted for optimization and enhancement, configured as a value-added service, or treated as a moderate supplement. This provides an intuitive basis for decision-making regarding the service portfolio and resource allocation for physical fitness promotion among older adults with chronic diseases.

Furthermore, based on the priority principle of “M > O > A > I” derived from the Kano model, the priority allocation order for physical fitness promotion services for older adults with chronic diseases was determined (as shown in Table 8).

**Table 8:** Priority ranking of need indicators by Kano attribute category among older adults with chronic diseases.

Need attribute	Priority ranking of need indicators
Must-be attribute	Scientific exercise guidance > Personalized exercise prescription > Routine physical fitness assessment > Physical fitness risk alerts > Periodic reassessment > Physical fitness risk grading > Establishment of personal health records > Interpretation of physical fitness risks
One-dimensional attribute	Healthy lifestyle guidance > Organization of social activities
Attractive attribute	Diverse testing venues > Age-friendly testing indicators > Medical follow-up > Family involvement
Indifferent attribute	Family rehabilitation training > Synchronization of physical fitness risks

In summary, compared with healthy older adults, the need structure of older adults with chronic diseases appears to place greater emphasis on health security than on experience enhancement. Specifically, services such as “scientific exercise guidance”, “personalized exercise prescription”, and “periodic reassessment” are classified as must-be attributes, forming the cornerstone of their physical fitness promotion. The absence of such services may lead to substantial dissatisfaction.

### 4.3 Physical fitness promotion needs of older adults with care dependency

#### 4.3.1 Overall hierarchical structure of needs among older adults with care dependency

Due to the compounding effects of physical functional limitations, reduced activity radius, and disease burden, the physical fitness promotion needs of older adults with care dependency exhibit differences from those of healthy older adults and older adults with chronic diseases in terms of both priority order and content composition. Based on the attribute classification results derived from the Kano model (as shown in Table 9), the need structure of this group is characterized by a high concentration of must-be attributes, accompanied by a small number of one-dimensional and attractive attributes.

**Table 9:** Kano attribute classification of physical fitness promotion needs among older adults with care dependency.

Function/service	A	O	M	I	R	Q	Kano classification
Establishment of personal health records	14.77%	12.50%	46.59%	21.59%	4.55%	0.00%	M
Routine physical fitness assessment	9.09%	14.77%	52.28%	23.86%	0.00%	0.00%	M
Diverse testing venues	17.05%	5.68%	13.64%	34.09%	27.27%	2.27%	I
Age-friendly testing indicators	18.18%	5.68%	30.68%	42.05%	3.41%	0.00%	I
Physical fitness risk alerts	10.23%	11.36%	40.91%	26.14%	11.36%	0.00%	M
Interpretation of physical fitness risks	11.36%	13.64%	42.05%	23.86%	9.09%	0.00%	M
Physical fitness risk grading	45.47%	11.36%	9.08%	32.95%	0.00%	1.14%	A
Synchronization of physical fitness risks	18.18%	10.23%	35.23%	21.59%	14.77%	0.00%	M
Personalized exercise prescription	12.50%	11.36%	48.87%	19.32%	7.95%	0.00%	M
Scientific exercise guidance	15.91%	14.77%	44.31%	17.05%	6.82%	1.14%	M
Healthy lifestyle guidance	17.05%	7.95%	60.23%	14.77%	0.00%	0.00%	M
Organization of social activities	22.73%	11.36%	21.59%	44.32%	0.00%	0.00%	I
Medical follow-up	17.05%	12.50%	53.40%	15.91%	1.14%	0.00%	M
Family rehabilitation training	18.18%	37.49%	17.05%	22.73%	3.41%	1.14%	O
Periodic reassessment	20.45%	14.77%	47.73%	17.05%	0.00%	0.00%	M
Family involvement	14.77%	19.32%	34.09%	18.18%	12.50%	1.14%	M

The results show that the vast majority of physical fitness promotion services constitute must-be needs for older adults with care dependency. These include the establishment of personal health records, routine physical fitness assessment, physical fitness risk alerts and interpretation, synchronization of physical fitness risks, personalized exercise prescription, scientific exercise guidance, healthy lifestyle guidance, medical follow-up, periodic health assessment, and family involvement. This finding is broadly consistent with previous research on the types of home- and community-based eldercare services required by older adults with care dependency (23). It suggests that this group has rigid needs for the integrity of health information systems, continuous monitoring of disease-related risks, individualized adaptation of rehabilitation exercise programs, and the involvement of multiple caregivers. These services may be regarded not only as minimum conditions for maintaining satisfaction, but also as potential supports for functional maintenance and risk management.

Among the one-dimensional attributes, only family rehabilitation training emerged as a prominent feature. This indicates that older adults with care dependency and their families are particularly sensitive to the acquisition of rehabilitation skills and expect that standardized rehabilitation methods will help improve their capacity for self-care. It may therefore be regarded as an important lever for enhancing satisfaction. At the same time, physical fitness risk grading displayed the characteristics of an attractive attribute, suggesting that even under conditions of functional limitation, older adults with care dependency still value a systematic understanding of their own health status and hope to receive individualized feedback and measures of progress (24). Its absence may not immediately generate strong dissatisfaction, but better evaluation and feedback services may help enhance their perceived value of the service.

By contrast, services such as diverse testing venues, age-friendly testing indicators, and organization of social activities were mostly classified as indifferent attributes. This may be related to the restricted mobility of this group, the narrowing of their living space, and the substantial increase in the cost of participation.

Overall, the service structure of older adults with care dependency exhibits a pronounced hierarchical pattern characterized by a predominance of basic rigid needs. In practice, priority may be given to ensuring the stable, continuous, and uninterrupted provision of must-be attributes. Family rehabilitation training may be treated as a key lever for improving the quality of family care, while attractive services such as physical fitness risk grading may be moderately strengthened where conditions permit, so as to enhance perceived service gains and subjective wellbeing without increasing burden.

#### 4.3.2 Satisfaction-dissatisfaction coefficient analysis of physical fitness promotion needs among older adults with care dependency

To identify the key service priorities and pathways for optimizing the service system structure in physical fitness promotion for older adults with care dependency, further analysis was conducted based on satisfaction and dissatisfaction coefficients (as shown in Table 10).

**Table 10:** Satisfaction–dissatisfaction coefficients of physical fitness promotion needs among older adults with care dependency.

Service	Attribute	Satisfaction coefficient	Dissatisfaction coefficient	Sensitivity
Establishment of personal health records	M	0.259	−0.642	0.692
Routine physical fitness assessment	M	0.244	−0.663	0.706
Diverse testing venues	I	0.339	−0.288	0.445
Age-friendly testing indicators	I	0.253	−0.386	0.461
Physical fitness risk alerts	M	0.250	−0.579	0.631
Interpretation of physical fitness risks	M	0.282	−0.603	0.665
Physical fitness risk grading	A	0.565	−0.212	0.603
Synchronization of physical fitness risks	M	0.347	−0.514	0.620
Personalized exercise prescription	M	0.259	−0.654	0.704
Scientific exercise guidance	M	0.342	−0.633	0.719
Healthy lifestyle guidance	M	0.273	−0.658	0.724
Organization of social activities	I	0.353	−0.341	0.491
Medical follow-up	M	0.306	−0.659	0.726
Family rehabilitation training	O	0.573	−0.561	0.802
Periodic reassessment	M	0.356	−0.621	0.716
Family involvement	M	0.405	−0.608	0.731

**Figure 5:**
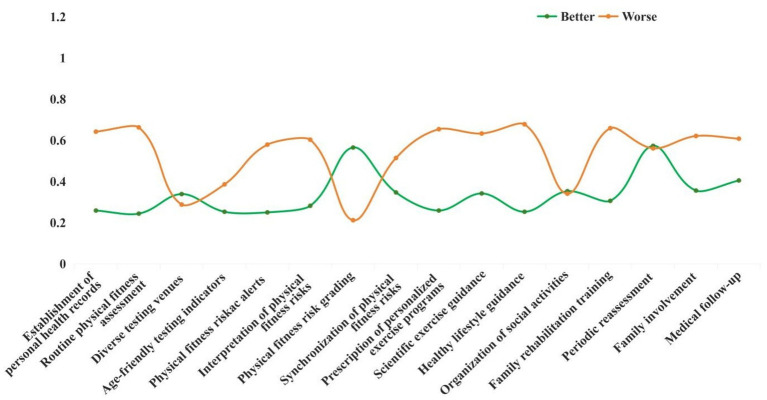
Satisfaction–dissatisfaction coefficients of each service item for older adults with care dependency.

As shown in Figure 5, the overall satisfaction–dissatisfaction curve for older adults with care dependency displays the typical pattern of greater dependency than expectation. This reflects their strong reliance on professional support, as their responses may be more closely associated with basic functional maintenance, safety support, continuity of care, and quality-of-life preservation, rather than with additional gains in satisfaction.

More specifically, the three services with the highest satisfaction coefficients were family rehabilitation training (0.573), physical fitness risk grading (0.565), and family involvement (0.405). Family rehabilitation training and family involvement jointly point to a family-centered model of collaborative care, aimed at creating a rehabilitation environment characterized by emotional support for older adults with care dependency. Physical fitness risk grading, by providing a clear reference point for health status and progress, enhances these older adults’ sense of perceptibility and controllability over their own condition, thereby playing an important role in sustaining rehabilitation motivation and may help improve their perceived understanding and control of functional changes. The dissatisfaction coefficients indicate that the absence of services such as routine physical fitness assessment (−0.663), medical follow-up (−0.659), and healthy lifestyle guidance (−0.658) would have a substantial negative impact on satisfaction, making them baseline elements for safeguarding the health and safety of older adults with care dependency.

**Figure 6:**
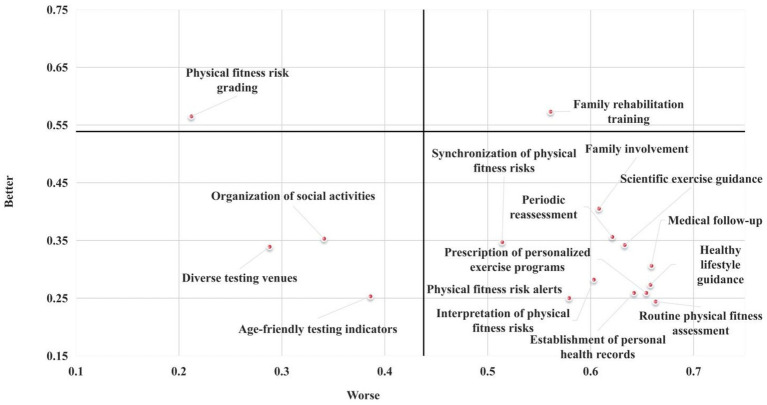
Satisfaction–dissatisfaction coefficient matrix of physical fitness promotion service needs among older adults with care dependency.

When interpreted in conjunction with the two-dimensional satisfaction–dissatisfaction matrix (see Figures 4–6), the sensitivity distribution and priority ranking of different service components can be visualized more clearly. Family rehabilitation training occupies a distinct position in the upper-right quadrant and ranks first in sensitivity (*S* = 0.802, satisfaction = 0.573, dissatisfaction = −0.561), indicating that it has a substantial effect on satisfaction under both enhanced provision and service absence. It is therefore a typical high-sensitivity one-dimensional attribute, underscoring its importance as a central lever for physical fitness promotion among older adults with care dependency. By comparison, must-be attributes such as family involvement (*S* = 0.731) and medical follow-up (*S* = 0.726) also display high sensitivity, and their bidirectional influence on satisfaction highlights the value of family support and professional care in maintaining the quality of life of this group. Services such as healthy lifestyle guidance, scientific exercise guidance, and periodic reassessment also rank among the top six in sensitivity, indicating that continuous health management and individualized exercise rehabilitation programs are critical components of a secure baseline. Overall, physical fitness promotion for older adults with care dependency may be organized around highly sensitive service elements, with priority given to ensuring the accessibility and continuity of those components that demonstrate strong sensitivity under both provision and absence scenarios.

Based on the attribute classification of the Kano model and the priority principle of “M > O > A > I,” combined with the sensitivity values derived from the analysis, the priority allocation order for physical fitness promotion services for older adults with care dependency was determined (as shown in Table 11).

**Table 11:** Priority ranking of need indicators by kano attribute category among older adults with care dependency.

Need attribute	Priority ranking of need indicators
Must-be attribute	Family involvement > Medical follow-up > Healthy lifestyle guidance > Scientific exercise guidance > Periodic reassessment > Routine physical fitness assessment > Personalized exercise prescription > Establishment of personal health records > Interpretation of physical fitness risks > Physical fitness risk alerts > Synchronization of physical fitness risks
One-dimensional attribute	Home rehabilitation training
Attractive attribute	Physical fitness risk grading
Indifferent attribute	Organization of social activities > Age-friendly testing indicators > Diverse testing venues

Overall, the physical fitness promotion needs of older adults with care dependency were more concentrated in basic guarantee and care-coordination services, especially showing higher sensitivity to medical follow-up, family involvement, healthy lifestyle guidance, scientific exercise guidance, and periodic reassessment. These findings may suggest that older adults with functional limitations place greater emphasis on safety support, continuity of care, and family coordination in physical fitness promotion services. At the same time, family-centered collaborative care appears to be an important support point for health promotion in this group. Therefore, relevant service strategies may prioritize the seamless provision of highly sensitive must-be services while ensuring basic safety. They may also focus on empowering families and strengthening support, so as to develop a physical fitness support system based on professional assistance and centered on family involvement.

## 5 Construction of a needs map for physical fitness promotion among older adults in urban communities

The preceding research has systematically identified the differentiated physical fitness promotion needs of healthy older adults, older adults with chronic diseases, and older adults with care dependency, determined the need attribute types of each service element, and characterized the marginal effects and fluctuation intensity of different elements under the scenarios of “enhanced supply—increased satisfaction” and “supply absence—exacerbated dissatisfaction.” On this basis, by integrating the closed-loop service chain of “monitoring—early warning—intervention—feedback” for physical fitness, the service elements were consolidated according to their functional stages to construct a needs map for physical fitness promotion among older adults in urban communities, providing a needs-based reference for subsequent health promotion services.

### 5.1 Physical fitness monitoring component

#### 5.1.1 Establishment of personal health records

The establishment of personal health records was identified as a must-be attribute across all three groups. The absolute values of the dissatisfaction coefficients associated with its absence were relatively high among healthy older adults (−0.644), older adults with chronic diseases (−0.637), and older adults with care dependency (−0.642). This suggests that the absence of personal health records may weaken service continuity and reduce trust and satisfaction. Functionally, the establishment of personal health records may not be understood merely as a tool for information storage, but rather as a potential data entry point for a closed-loop service system. In addition to basic demographic information and medical history, such records may integrate multi-source data, including physical fitness assessment results, exercise intervention records, lifestyle information, and social support information. Through information platforms, these data could be collected and standardized to form individual profiles that may support physical fitness risk alerts and intervention adjustment. At the same time, personal health records may be assigned unique identifiers to support cross-institutional access and dynamic updating. Under the premise of graded authorization and privacy protection, relevant information could be shared with necessary service providers, such as community health service centers, physical fitness and health centers, and day-care centers, thereby providing a data foundation for closed-loop physical fitness promotion services.

#### 5.1.2 Routine physical fitness assessment

Routine physical fitness assessment was also identified as a must-be attribute across all three groups. The absolute values of the dissatisfaction coefficients were relatively high among healthy older adults (−0.716), older adults with chronic diseases (−0.710), and older adults with care dependency (−0.663). This suggests that interruptions in assessment provision or declines in assessment quality may lead to notable dissatisfaction. At the same time, diversified assessment sites, as a service carrier for routine physical fitness assessment, showed clear experience-enhancing value among healthy older adults and older adults with chronic diseases. The satisfaction coefficients for this item were relatively high in both groups (0.692 among healthy older adults and 0.702 among older adults with chronic diseases), while the absolute values of the dissatisfaction coefficients were relatively low (−0.227 and −0.209, respectively), indicating a value-added attribute. Taken together, these findings suggest that physical fitness assessment itself represents a basic service component, whereas diversification of assessment sites may further improve satisfaction among healthy older adults and older adults with chronic diseases. Accordingly, for healthy older adults and older adults with chronic diseases, routine physical fitness assessment could be supported by frequently used community spaces, such as community health service centers, day-care centers, cultural activity stations, physical fitness and health centers, and senior dining facilities. Smart physical fitness monitoring devices may be reasonably deployed in these settings to support the development of a routine monitoring network. For older adults with care dependency, home-based assessment may be more appropriate, taking into account spatial accessibility and family care conditions, so as to support continuous monitoring without adding unnecessary burden.

### 5.2 Physical fitness early warning component

#### 5.2.1 Physical fitness risk alerts

The physical fitness risk alert service was classified as a one-dimensional attribute among healthy older adults and ranked first in sensitivity (satisfaction coefficient = 0.645; dissatisfaction coefficient = −0.660; *S* = 0.923). This indicates that it may serve both as an important source of satisfaction improvement and as a major trigger of dissatisfaction when absent. Among older adults with chronic diseases and those with care dependency, the physical fitness risk alert service was classified as a must-be attribute (older adults with chronic diseases: satisfaction coefficient = 0.330, dissatisfaction coefficient = −0.646, *S* = 0.725; older adults with care dependency: satisfaction coefficient = 0.250, dissatisfaction coefficient = −0.579, *S* = 0.631), suggesting that it may already be perceived as a basic component of physical fitness promotion services. Accordingly, in the process of providing physical fitness promotion services, it may be useful to conduct a comprehensive assessment of key physical fitness indicators and their changes based on monitoring results, generate graded and classified risk messages, and deliver them to older adults in the community through multiple channels, such as telephone calls and text messages.

#### 5.2.2 Interpretation of physical fitness risks

Among healthy older adults, interpretation of physical fitness risks was likewise classified as a one-dimensional attribute, and it had the highest absolute dissatisfaction coefficient (satisfaction coefficient = 0.592, dissatisfaction coefficient = −0.691, *S* = 0.910). This indicates that risk alerts without corresponding interpretation are likely to amplify dissatisfaction substantially. By contrast, among older adults with chronic diseases and those with care dependency, interpretation of physical fitness risks was classified as a must-be attribute (chronic disease group: satisfaction coefficient = 0.292, dissatisfaction coefficient = −0.540, *S* = 0.614; care dependency group: satisfaction coefficient = 0.282, dissatisfaction coefficient = −0.603, *S* = 0.665), making it an important anchor of continuous risk management. For this reason, the physical fitness early warning process may not only communicate the existence of risk but also explain the meaning of the physical fitness indicators, the mechanisms through which the risks arise, and their possible consequences, thereby guiding older adults’ subsequent actions. In implementation, different forms of communication may be adopted according to educational level and health literacy, including visual presentation, tiered written explanations, and case-based interpretation, so as to avoid the indiscriminate use of technical jargon. For older adults who live alone and have relatively limited emotional support, the tone of explanation may also be reassuring, in order to reduce anxiety arising from information asymmetry.

#### 5.2.3 Physical fitness risk grading

Physical fitness risk grading was classified as a one-dimensional attribute among healthy older adults (satisfaction coefficient = 0.634; dissatisfaction coefficient = −0.623; *S* = 0.889), indicating a close association with overall service satisfaction. Among older adults with chronic diseases, it was classified as a must-be attribute (satisfaction coefficient = 0.321; dissatisfaction coefficient = −0.636; *S* = 0.712), suggesting that it had become a basic component supporting physical fitness promotion. Among older adults with care dependency, it showed the characteristics of an attractive attribute (satisfaction coefficient = 0.565; dissatisfaction coefficient = −0.212; *S* = 0.603), indicating that well-delivered evaluation results may enhance their sense of service gain and confidence in rehabilitation. Accordingly, physical fitness risk grading could provide a comprehensive conclusion on the basis of physical fitness risk alerts and interpretation of physical fitness risks. Relevant data may also be incorporated into personal health records to inform subsequent intervention and feedback. In practice, the content and delivery of evaluation feedback may be stratified. For healthy older adults, feedback may emphasize trend comparisons with peers of the same age group. For older adults with chronic diseases, physical fitness risk grading may be closely linked to disease control indicators and functional changes. For older adults with care dependency, feedback may appropriately highlight the positive significance of functional improvement and be delivered through family members or caregivers when necessary, so as to strengthen their confidence in participating in physical fitness promotion.

### 5.3 Physical fitness intervention component

#### 5.3.1 Personalized exercise prescription

Personalized exercise prescription was identified as a must-be attribute among healthy older adults, older adults with chronic diseases, and older adults with care dependency. The absolute values of the dissatisfaction coefficients were relatively high across the three groups (healthy older adults: −0.659; older adults with chronic diseases: −0.728; older adults with care dependency: −0.654), suggesting that personalized exercise prescription represents both a baseline component of physical fitness intervention and a key factor associated with overall satisfaction. Accordingly, in service design, personalized exercise prescription may be developed for community-dwelling older adults based on physical fitness monitoring and physical fitness risk alerts results, with reference to their personal health records. Such prescriptions could specify the type, intensity, frequency, duration, and precautions of exercise, thereby providing an operational basis for safe and effective physical fitness intervention.

#### 5.3.2 Scientific exercise guidance

Scientific exercise guidance showed typical attractive-attribute characteristics among healthy older adults (satisfaction coefficient = 0.698; dissatisfaction coefficient = −0.238; *S* = 0.738), indicating that it is an important factor for improving service experience. Among older adults with chronic diseases and those with care dependency, it was identified as a must-be attribute (older adults with chronic diseases: satisfaction coefficient = 0.326, dissatisfaction coefficient = −0.692, *S* = 0.765; older adults with care dependency: satisfaction coefficient = 0.342, dissatisfaction coefficient = −0.633, *S* = 0.719). This suggests that insufficient provision of scientific exercise guidance may not only weaken the effectiveness of intervention but also reduce overall satisfaction. Therefore, after personalized exercise prescriptions are developed, scientific exercise guidance may be incorporated as a core service component. Qualified exercise health professionals may provide stratified and tailored guidance based on the prescription content. For healthy older adults, intervention content may focus on maintaining physical function and improving health literacy. For older adults with chronic diseases, guidance may aim to maintain physical function while supporting chronic disease rehabilitation. For older adults with care dependency, intervention content may emphasize rehabilitation orientation and maintenance of daily living ability, thereby forming an intervention framework centered on functional activation, capacity restoration, and family support.

#### 5.3.3 Healthy lifestyle guidance

Healthy lifestyle guidance was identified as an attractive attribute among healthy older adults (satisfaction coefficient = 0.527; dissatisfaction coefficient = −0.307; *S* = 0.610), suggesting that it may contribute to satisfaction improvement. Among older adults with chronic diseases, it was classified as a one-dimensional attribute and had the highest sensitivity score (satisfaction coefficient = 0.606; dissatisfaction coefficient = −0.640; *S* = 0.882), indicating that it is an important factor associated with their satisfaction. Among older adults with care dependency, it was classified as a must-be attribute (satisfaction coefficient = 0.273; dissatisfaction coefficient = −0.658; *S* = 0.724), suggesting that the absence of this service may substantially reduce satisfaction. Accordingly, healthy lifestyle guidance may be embedded throughout the physical fitness intervention process. It could provide older adults with systematic intervention plans around four core dimensions: scientific diet, regular sleep, psychological stability, and appropriate health behaviors.

#### 5.3.4 Organization of social activities

Organization of social activities was identified as a one-dimensional attribute among both healthy older adults and older adults with chronic diseases (healthy older adults: satisfaction coefficient = 0.590, dissatisfaction coefficient = −0.646, *S* = 0.875; older adults with chronic diseases: satisfaction coefficient = 0.590, dissatisfaction coefficient = −0.646, *S* = 0.875). This suggests that well-designed social activities may substantially improve satisfaction, whereas their absence may lead to considerable dissatisfaction. In contrast, older adults with care dependency showed relatively lower sensitivity to organization of social activities, which was classified as an indifferent attribute (satisfaction coefficient = 0.353; dissatisfaction coefficient = −0.341; *S* = 0.491). This may indicate that their primary concerns are more closely related to safety assurance and care support, while the marginal perceived value of social participation is relatively limited. Accordingly, organization of social activities may be incorporated into the physical fitness intervention process. For healthy older adults and older adults with chronic diseases, activities such as group fitness exercises, collective Tai Chi, recreational sports events, and interest groups may integrate physical activity with peer interaction, thereby supporting both physical function and emotional communication needs. For older adults with care dependency, although social activities were not highly sensitive in the Kano classification, small-scale communication activities centered on emotional support may still be moderately embedded in family care settings as an auxiliary intervention approach.

#### 5.3.5 Medical follow-up, family rehabilitation training, and family involvement

Medical follow-up (satisfaction coefficient = 0.405; dissatisfaction coefficient = −0.203; *S* = 0.453) and family rehabilitation training (satisfaction coefficient = 0.211; dissatisfaction coefficient = −0.358; *S* = 0.415) were classified as indifferent attributes among healthy older adults, while family involvement was identified as a reverse attribute (satisfaction coefficient = 0.228; dissatisfaction coefficient = −0.287; *S* = 0.366). This may reflect a stronger emphasis on autonomy and a reluctance to increase the caregiving burden on adult children within this group. Accordingly, the presence or absence of these services appears to have relatively limited influence on overall satisfaction and, in some cases, may even be perceived negatively. Among older adults with chronic diseases, medical follow-up (satisfaction coefficient = 0.679; dissatisfaction coefficient = −0.146; *S* = 0.695) and family involvement (satisfaction coefficient = 0.541; dissatisfaction coefficient = −0.317; *S* = 0.627) were identified as attractive attributes. Family rehabilitation training (satisfaction coefficient = 0.406; dissatisfaction coefficient = −0.460; *S* = 0.613) was classified as an indifferent attribute, although the relatively high absolute value of its dissatisfaction coefficient suggests that high-quality follow-up support and rehabilitation guidance may help improve satisfaction among older adults with chronic diseases. For older adults with care dependency, medical follow-up (satisfaction coefficient = 0.306; dissatisfaction coefficient = −0.659; *S* = 0.726) and family involvement (satisfaction coefficient = 0.405; dissatisfaction coefficient = −0.608; *S* = 0.731) were both high-sensitivity must-be attributes. Family rehabilitation training (satisfaction coefficient = 0.573; dissatisfaction coefficient = −0.561; *S* = 0.802) was identified as a high-sensitivity one-dimensional service, highlighting the potential importance of home-based coordination for safety support and functional maintenance. Based on these differences, physical fitness intervention services for older adults with care dependency may include an integrated service package consisting of medical follow-up, family rehabilitation training, and family involvement, with follow-up frequency and guidance content adjusted according to risk level. For older adults with chronic diseases, these three services may be configured as key value-added modules to enhance satisfaction. For healthy older adults, mandatory family involvement may be reduced, and their autonomy may be fully respected.

### 5.4 Physical fitness feedback component

#### 5.4.1 Periodic reassessment and feedback

Periodic reassessment was identified as a must-be attribute across all three groups. The absolute values of the dissatisfaction coefficients were relatively high among healthy older adults (−0.640), older adults with chronic diseases (−0.685), and older adults with care dependency (−0.621), and the sensitivity scores also remained at relatively high levels (0.694, 0.712, and 0.716, respectively). This suggests that the absence of periodic reassessment may not only weaken older adults’ perceived effectiveness of interventions, but also reduce their trust in the overall service system. Accordingly, in the feedback stage of physical fitness promotion, periodic reassessment may be regarded as a core component. A comprehensive physical fitness reassessment could be conducted quarterly to longitudinally track key physical fitness indicators, exercise adherence, lifestyle changes, and perceived health status. For older adults with chronic diseases and those with care dependency, the evaluation interval may be appropriately shortened according to risk level and functional changes. At the same time, reassessment results may be promptly incorporated into personal health records and fed back to older adults through visualized formats such as charts, thereby supporting their willingness to participate in physical fitness promotion. These data may also provide a decision-making basis for relevant service institutions to optimize and refine services, thereby supporting the closed-loop operation of physical fitness promotion services.

## 6 Implications for meeting the needs for physical fitness promotion among older adults in urban communities

### 6.1 Prioritizing service provision according to need attributes

Physical fitness promotion service needs among older adults in urban communities show certain hierarchical differences, and different service components occupy different positions within the need structure. In practice, constraints related to resources, professional capacity, and service delivery may make it difficult to develop all services simultaneously and evenly. Therefore, service priorities may be differentiated according to need attributes, with foundational services addressed first and enhancement-oriented services gradually improved thereafter. Based on the preceding analysis, must-be attributes are closer to the baseline requirements of the service system. Their absence is more likely to affect older adults’ basic evaluations of the service and may therefore be prioritized within basic service provision. One-dimensional attributes are more directly associated with improvements in service perception and may be regarded as key areas for further enhancement once basic services are in place. Attractive attributes do not constitute necessary conditions for maintaining service operation, but they may play a positive role in strengthening participation willingness and perceived service gain, and may therefore be gradually introduced according to local community conditions. For physical fitness promotion among older adults in urban communities, service arrangements may first consolidate the supply foundation around basic needs that are common across groups. On this basis, priorities may be adjusted according to differences in health status, functional level, and care dependency among different groups of older adults, thereby avoiding undifferentiated investment and homogeneous service provision.

### 6.2 Tailoring service content to group differences

The survey results indicate that different types of older adults show certain differences in their physical fitness promotion needs. Therefore, the arrangement of service content may take into account the actual circumstances and priority needs of different groups. For healthy older adults, service priorities may focus on physical fitness monitoring, risk identification, exercise guidance, health education, and social participation, with greater emphasis on preventing physical fitness decline, maintaining physical function, and promoting active participation. For older adults with chronic diseases, service content may need to go beyond general health promotion. Greater attention may be given to the linkage among monitoring, physical fitness risk alerts, intervention, and follow-up, with risk control, exercise rehabilitation, and lifestyle adjustment incorporated into a continuous management process. For older adults with care dependency, the service focus may further shift toward functional maintenance, daily support, and care coordination, with greater attention to medical follow-up, family rehabilitation training, and family involvement. Overall, service content configuration may not be developed in a uniform manner without considering the physical conditions and living circumstances of different groups of older adults. Nor could a single service standard be applied mechanically to all groups. Rather, on the premise that common basic services are ensured, service arrangements may be adjusted with emphasis on the most prominent practical needs of each group.

### 6.3 Improving channels for need expression to promote effective service provision

In the service process, older adults are often positioned as passive recipients. Although their needs exist objectively, the lack of stable and effective channels for expressing those needs may lead to a mismatch between service provision and actual need. Therefore, future practice may consider further improving channels for need expression and feedback, so as to enhance the accuracy of service identification and supply alignment. On the one hand, information on older adults’ physical fitness promotion needs may be continuously collected through questionnaires, home visits, resident discussions, and health record tracking, thereby improving the comprehensiveness of need identification. On the other hand, dispersed individual needs may be organized and summarized, gradually transforming them into important references for community service arrangements, program design, and resource allocation. At the same time, attention may also be given to differences in expression ability, participation conditions, and types of needs among different groups. For healthy older adults, feedback may be collected through routine participation channels. For older adults with chronic diseases, need information may be gathered in combination with health management and follow-up processes. For older adults with care dependency, family members, community workers, and professionals may play a greater role in assisting with need communication.

## 7 Conclusion

Based on the Kano model, this study classified and hierarchically analyzed physical fitness promotion needs among older adults recruited from urban community service-related settings. The findings indicate that the physical fitness promotion service needs of the three groups of older adults showed different Kano attribute distribution patterns. The needs of healthy older adults were characterized by both basic service guarantees and health information support. The needs of older adults with chronic diseases were more concentrated in continuous support services, such as routine physical fitness assessment, physical fitness risk alerts, personalized exercise prescription, and scientific exercise guidance. The needs of older adults with care dependency further clustered around care-coordination services, including medical follow-up, family involvement, healthy lifestyle guidance, and family rehabilitation training. Overall, older adults with different health conditions and functional statuses may have stratified perceived attributes and priority needs for physical fitness promotion services. This suggests that subsequent service provision could not adopt a uniform allocation approach, but may instead be designed in a stratified manner according to older adults’ health status, physical function, and level of care dependency.

This study also has several limitations. First, this study used a modified Kano questionnaire. Although the questionnaire retained the paired structure of functional and dysfunctional questions and classified service attributes according to the Kano evaluation matrix, the response options adopted a satisfaction-based format ranging from “very satisfied” to “very dissatisfied,” rather than the preference-based options commonly used in the classic Kano questionnaire. This adaptation may help reduce the difficulty of comprehension for older respondents, but it may also affect their judgment of the dysfunctional questions. Because this study did not conduct systematic cognitive interviews or comparative testing of different response formats, the applicability of the modified Kano questionnaire requires further validation. Meanwhile, the Kano model can identify service need attributes and priority levels, but it cannot explain the causal mechanisms underlying these needs, nor can it evaluate the actual effectiveness of service optimization strategies. Second, the study sample was recruited from various urban community service-related sites in Shanghai, Zhengzhou, and Chengdu. It was not a strictly probability-based sample and may not be regarded as representative of all community-dwelling older adults in urban areas. Therefore, the findings are mainly applicable to understanding the structure of physical fitness promotion service needs among older respondents in urban community service-related settings, and may not be directly generalized to all urban older adults. In particular, a relatively large proportion of older adults with care dependency were recruited from eldercare institutions, day-care centers, and hospitals, which may have influenced their service need evaluations and Kano attribute classifications. Third, the sample size of older adults with care dependency was relatively small, with only 88 participants included. Because this group is less accessible in field surveys, and because the Kano model classifies attributes primarily based on the category with the highest proportion, the attribute classification of some service items in this subgroup may be unstable under small-sample conditions. Therefore, the findings for older adults with care dependency may be regarded as exploratory, mainly indicating possible tendencies in their service need attributes, and may not be overgeneralized. Fourth, this study mainly identified the perceived attributes and priority structure of physical fitness promotion service needs among the respondents, but did not conduct actual intervention testing or effectiveness evaluation of the proposed service optimization directions. Therefore, the related service recommendations may be understood as practical implications derived from need identification, rather than institutional solutions or service models directly validated by the data in this study. Future research may further verify the applicability of the questionnaire, the stability of need classification, and the actual effectiveness of service optimization pathways by expanding the sample size, improving sampling design, conducting cognitive interviews, and using longitudinal tracking or intervention studies.

## Data Availability

The original contributions presented in the study are included in the article/Supplementary material, further inquiries can be directed to the corresponding author.
