# Supplementary material for: Hierarchical service needs for physical fitness promotion among Urban Community older adults: a Kano model perspective
Source: Front Public Health. 2026 Jun 22;14:1843368. doi: 10.3389/fpubh.2026.1843368 (PMC13333349; doi:10.3389/fpubh.2026.1843368)
Supplement: Supplementary file 1 [file Supplementary_File_1.pdf]

Supplementary File 1. Delphi Expert Consultation Results Report

1. Basic Information of the Expert Consultation

This study employed a two-round Delphi expert consultation to screen and revise the indicator system of physical fitness promotion service needs for older adults in urban communities. The first round of expert consultation focused on evaluating the initial indicator pool, with particular emphasis on the importance, relevance, comprehensibility, and operability of the indicators. Based on the revisions made after the first round, the second round of consultation was conducted primarily to confirm the stability of the revised indicator system and the degree of consensus among the experts.

A total of 10 experts were invited to participate in the consultation. The experts came from the fields of sports science, public health, geriatric medicine, and community health services, and all possessed substantial theoretical research or practical work experience.

The expert inclusion criteria were: holding a senior professional title (associate professor or above) or a doctoral degree; having at least five years of experience in research or practice related to health promotion for older adults, community health services, exercise intervention, or related fields; being familiar with physical fitness promotion, health management for older adults, or community service provision; and being able to independently complete the expert consultation questionnaire and provide substantive comments for revision.

The expert response rates, authority coefficients, and degrees of consensus in the two Delphi rounds are presented in Tables S1 and S2.

Table S1 Expert Response Rate and Authority Coefficient

Round	Questionnaires Distributed	Questionnaires Returned	Effective Response Rate	Judgment Basis Coefficient	Familiarity Coefficient	Expert Authority Coefficient
First	10	10	100.00%	0.86	0.82	0.84
Second	10	9	90.00%	0.88	0.84	0.86

Note: The expert authority coefficient was calculated as the average of the judgment basis coefficient and the familiarity coefficient. It is generally accepted that an expert authority coefficient greater than 0.70 indicates a high level of credibility of the expert consultation results.

Table S2 Kendall's Coefficient of Concordance Test Results

Round	Number of Experts	Number of Indicators	Kendall's W	Chi-square (χ^2)	Degrees of Freedom	p
First	10	24	0.286	65.780	23	<0.001
Second	9	16	0.436	58.860	15	<0.001

Note: Kendall's coefficient of concordance (W) is used to test the consistency of expert opinions. The p-values for both rounds were less than 0.001, indicating that the degree of expert consensus was statistically significant. The higher Kendall's W in the second round compared to the first round indicates that after the first round of revisions, expert opinions became further concentrated.

2. First Round Delphi Expert Consultation Results: Evaluation of the Initial Indicator Pool

The first round of expert consultation focused on the initial indicator pool. The initial indicator pool was developed based on literature review, practical case analysis, and research team discussions. It comprised 4 first-level dimensions and 24 second-level indicators. Specifically, the physical fitness monitoring dimension had 6 items, the physical fitness early warning dimension had 6 items, the physical fitness intervention dimension had 8 items, and the physical fitness feedback dimension had 4 items. Experts evaluated the indicators primarily in terms of importance, relevance, comprehensibility, and operability, and provided open-ended comments for revision.

The indicator retention criteria were set as: mean importance score ≥ 4.00, coefficient of variation ≤ 0.25, and full-score ratio ≥ 0.20. Indicators that did not meet these criteria, or that met them but exhibited conceptual overlap, unclear boundaries, or non-standard wording, were deleted, merged, renamed, or revised based on expert feedback.

Table S3 First Round Delphi Expert Consultation Results: Evaluation of the Initial Indicator Pool

First-Level Dimension	Initial Indicator	Mean	CV	Full-score ratio	First-Round Handling Decision
Physical Fitness Monitoring	Basic health information collection	4.20	0.173	0.300	Merged with "Establishment of personal physical fitness records"
Physical Fitness Monitoring	Establishment of personal physical fitness records	4.60	0.112	0.600	Merged and renamed "Establishment of personal health records"
Physical Fitness Monitoring	Routine physical fitness testing	4.70	0.101	0.700	Renamed "Routine physical fitness assessment"
Physical Fitness Monitoring	Diverse testing venues	4.40	0.159	0.500	Retained, wording revised
Physical Fitness Monitoring	Age-friendly testing indicators	4.50	0.117	0.500	Retained, connotation of age-appropriateness supplemented
Physical Fitness Monitoring	Smart wearable device monitoring	3.80	0.263	0.200	Deleted; experts considered accessibility and generalizability inadequate
Physical Fitness Early Warning	Physical fitness risk alerts	4.70	0.101	0.700	Retained, integrated specific risk content
Physical Fitness Early Warning	Interpretation of physical fitness risks	4.50	0.117	0.500	Retained, emphasized professional interpretation
Physical Fitness Early Warning	Physical fitness risk grading	4.60	0.112	0.600	Retained, linked to risk levels
Physical Fitness Early Warning	Synchronization of physical fitness risks	4.30	0.163	0.400	Retained, clarified target of synchronization
Physical Fitness Early Warning	Specialized screening for falls and sarcopenia	4.30	0.163	0.400	Merged into "Physical fitness risk alerts" and "Physical fitness risk grading"

First-Level Dimension	Initial Indicator	Mean	CV	Full-score ratio	First-Round Handling Decision
Physical Fitness Early Warning	Online early warning push notifications	3.90	0.256	0.200	Deleted; experts noted considerable variation in digital accessibility
Physical Fitness Intervention	Personalized exercise prescription	4.70	0.101	0.700	Retained, revised basis for prescription
Physical Fitness Intervention	Scientific exercise guidance	4.60	0.112	0.600	Retained, clarified implementing actor
Physical Fitness Intervention	Healthy lifestyle guidance	4.40	0.159	0.500	Retained, integrated dietary and psychological content
Physical Fitness Intervention	Nutritional dietary guidance	4.00	0.237	0.200	Merged into "Healthy lifestyle guidance"
Physical Fitness Intervention	Psychological adjustment guidance	3.90	0.247	0.200	Merged into "Healthy lifestyle guidance"
Physical Fitness Intervention	Organization of social activities	4.30	0.163	0.400	Retained, wording revised
Physical Fitness Intervention	Medical follow-up	4.50	0.117	0.500	Retained, clarified collaboration between medical staff and exercise health specialists
Physical Fitness Intervention	Family rehabilitation training	4.30	0.163	0.400	Retained, emphasized family caregiving capacity
Physical Fitness Feedback	Periodic reassessment	4.60	0.112	0.600	Retained, integrated feedback on effects
Physical Fitness Feedback	Intervention effect feedback report	4.20	0.181	0.300	Merged into "Periodic reassessment"
Physical Fitness Feedback	Family involvement	4.30	0.163	0.400	Retained, clarified involvement in feedback and planning discussions

First-Level Dimension	Initial Indicator	Mean	CV	Full-score ratio	First-Round Handling Decision
Physical Fitness Feedback	Online satisfaction feedback	3.70	0.287	0.100	Deleted; experts considered functional overlap with Kano satisfaction measurement

3. First-Round Indicator Revisions

Based on the first-round expert ratings and open-ended comments, the research team made the following adjustments to the initial indicator pool.

First, some indicators with inadequate accessibility or weak relevance to the research topic were deleted. These included "Smart wearable device monitoring," "Online early warning push notifications," and "Online satisfaction feedback." Experts noted that although these indicators have value in representing digital services, given that the study population primarily consists of older adults, there are significant differences in digital device usage capacity and service accessibility. Including them as core need indicators could reduce the generalizability of the indicator system.

Second, indicators with conceptual overlap or functional duplication were merged. "Basic health information collection" and "Establishment of personal physical fitness records" were merged into "Establishment of personal health records" to highlight the dynamic updating and data integration functions of the records. "Specialized screening for falls and sarcopenia" was merged into "Physical fitness risk alerts" and "Physical fitness risk grading" to avoid excessive fragmentation of indicators within the early warning dimension. "Nutritional dietary guidance" and "Psychological adjustment guidance" were merged into "Healthy lifestyle guidance" to form a more comprehensive lifestyle intervention indicator. "Intervention effect feedback report" was merged into "Periodic reassessment" to emphasize the functions of re-evaluation, comparison, and program adjustment in the feedback loop.

Third, some indicators were renamed or had their wording revised. "Routine physical fitness testing" was revised to "Routine physical fitness assessment" to better reflect the interpretation of test results, judgment of functional status, and linkage to subsequent services. "Establishment of personal physical fitness records" was revised to "Establishment of personal health records" to encompass physical fitness data, health information, and service records. For indicators such as "Synchronization of physical fitness risks," "Medical follow-up," "Family rehabilitation training," and "Family involvement," the target population, implementing actor, and operational boundaries were further clarified.

Following the first-round revisions, the initial 24 indicators were adjusted into 4 dimensions with 16 indicators, forming the basis for the second-round expert consultation questionnaire.

4. Second Round Delphi Expert Consultation Results: Confirmation of the Revised Indicator System

The second round of expert consultation focused on the revised indicator system of 4 dimensions and 16 indicators, primarily examining the stability, completeness, and degree of expert consensus regarding the indicator system. The results showed that in the second round, the mean importance scores of the indicators ranged from 4.56 to 4.89, the coefficients of variation ranged from 0.068 to 0.133, and the full-score ratios ranged from 0.556 to 0.889, all meeting the pre-set retention criteria. Compared with the first round, the coefficients of variation generally decreased in the second round, and the degree of expert consensus improved, indicating that the revised indicator system possesses good content validity and face validity.

Table S4 Second Round Delphi Expert Consultation Results: Confirmation of the Revised Indicator System

First-Level Dimension	Revised Indicator	Mean	CV	Full-score ratio	Second-Round Handling Decision
Physical Fitness Monitoring	Establishment of personal health records	4.78	0.092	0.778	Retained
Physical Fitness Monitoring	Routine physical fitness assessment	4.89	0.068	0.889	Retained
Physical Fitness Monitoring	Diverse testing venues	4.67	0.107	0.667	Retained
Physical Fitness Monitoring	Age-friendly testing indicators	4.67	0.107	0.667	Retained
Physical Fitness Early Warning	Physical fitness risk alerts	4.89	0.068	0.889	Retained
Physical Fitness Early Warning	Interpretation of physical fitness risks	4.78	0.092	0.778	Retained
Physical Fitness Early Warning	Physical fitness risk grading	4.78	0.092	0.778	Retained
Physical Fitness Early Warning	Synchronization of physical fitness risks	4.56	0.117	0.556	Retained

First-Level Dimension	Revised Indicator	Mean	CV	Full-score ratio	Second-Round Handling Decision
Physical Fitness Intervention	Personalized exercise prescription	4.89	0.068	0.889	Retained
Physical Fitness Intervention	Scientific exercise guidance	4.78	0.092	0.778	Retained
Physical Fitness Intervention	Healthy lifestyle guidance	4.67	0.107	0.667	Retained
Physical Fitness Intervention	Organization of social activities	4.56	0.133	0.556	Retained
Physical Fitness Intervention	Medical follow-up	4.78	0.092	0.778	Retained
Physical Fitness Intervention	Family rehabilitation training	4.56	0.133	0.556	Retained
Physical Fitness Feedback	Periodic reassessment	4.78	0.092	0.778	Retained
Physical Fitness Feedback	Family involvement	4.56	0.133	0.556	Retained

Note: The full-score ratio refers to the proportion of experts assigning the highest score to an indicator. The coefficient of variation (CV) reflects the degree of dispersion of expert opinions. In the second round, all indicators met the retention criteria of mean ⩾ 4.00, CV ⩽ 0.25, and full-score ratio ⩾ 0.20.

5. Final Indicator System

Following the two rounds of expert consultation and research team discussions, the final indicator system for physical fitness promotion service needs of older adults in urban communities was established. It comprises 4 first-level dimensions (physical fitness monitoring, physical fitness early warning, physical fitness intervention, and physical fitness feedback) and 16 second-level indicators.

Table S5 Final Indicator System for Physical Fitness Promotion Service Needs of Older Adults in Urban Communities

First-Level Dimension	Second-Level Indicator	Indicator Description
Physical Fitness Monitoring	Establishment of personal health records	Establishing dynamically updated personal health records for older adults, integrating basic information, physical fitness data, health risks, and service records

First-Level Dimension	Second-Level Indicator	Indicator Description
Physical Fitness Monitoring	Routine physical fitness assessment	Regularly conducting standardized physical fitness testing and functional status assessment
Physical Fitness Monitoring	Diverse testing venues	Providing convenient testing services in settings such as community health service centers, day-care centers, and community physical fitness and health centers
Physical Fitness Monitoring	Age-friendly testing indicators	Adopting assessment items appropriate for the physical functional characteristics of older adults, avoiding high-intensity or high-risk tests
Physical Fitness Early Warning	Physical fitness risk alerts	Alerting on physical fitness risks based on information such as fitness monitoring results, chronic disease history, and lifestyle factors
Physical Fitness Early Warning	Interpretation of physical fitness risks	Having professionals explain the risk results, their causes, and coping strategies
Physical Fitness Early Warning	Physical fitness risk grading	Forming a graded physical fitness risk evaluation and providing corresponding tiered intervention recommendations
Physical Fitness Early Warning	Synchronization of physical fitness risks	Sharing necessary risk information with family members or caregivers to support home-based collaborative management
Physical Fitness Intervention	Personalized exercise prescription	Prescribing personalized exercise programs based on physical fitness assessment results and health risk levels
Physical Fitness Intervention	Scientific exercise guidance	Providing stratified, categorized guidance around exercise prescriptions by qualified exercise health specialists
Physical Fitness Intervention	Healthy lifestyle guidance	Offering comprehensive health guidance covering diet, sleep,

First-Level Dimension	Second-Level Indicator	Indicator Description
		psychological adjustment, and other aspects
Physical Fitness Intervention	Organization of social activities	Organizing group activities such as tai chi, square dancing, and group exercise to promote physical activity and social participation
Physical Fitness Intervention	Medical follow-up	Conducting health follow-ups and rehabilitation guidance by community general practitioners, nurses, or exercise health specialists
Physical Fitness Intervention	Family rehabilitation training	Providing family members with training on basic rehabilitation knowledge and caregiving skills
Physical Fitness Feedback	Periodic reassessment	Periodically reassessing physical fitness indicators, comparing changes before and after intervention, and adjusting plans accordingly
Physical Fitness Feedback	Family involvement	Inviting family members to participate in feedback discussions and the development of the next phase of exercise plans
